# RIG-I and MDA-5 Detection of Viral RNA-dependent RNA Polymerase Activity Restricts Positive-Strand RNA Virus Replication

**DOI:** 10.1371/journal.ppat.1003610

**Published:** 2013-09-05

**Authors:** Andrei Nikonov, Tarmo Mölder, Rein Sikut, Kaja Kiiver, Andres Männik, Urve Toots, Aleksei Lulla, Valeria Lulla, Age Utt, Andres Merits, Mart Ustav

**Affiliations:** 1 Department of Biomedical Technology, Institute of Technology, University of Tartu, Tartu, Estonia; 2 FIT Biotech Oy, Tartu, Estonia; 3 Icosagen Cell Factory OÜ, Tartu, Estonia; 4 Estonian Academy of Sciences, Tallinn, Estonia; North Carolina State University, United States of America

## Abstract

Type I interferons (IFN) are important for antiviral responses. Melanoma differentiation-associated gene 5 (MDA-5) and retinoic acid-induced gene I (RIG-I) proteins detect cytosolic double-stranded RNA (dsRNA) or 5′-triphosphate (5′-ppp) RNA and mediate IFN production. Cytosolic 5′-ppp RNA and dsRNA are generated during viral RNA replication and transcription by viral RNA replicases [RNA-dependent RNA polymerases (RdRp)]. Here, we show that the Semliki Forest virus (SFV) RNA replicase can induce IFN-β independently of viral RNA replication and transcription. The SFV replicase converts host cell RNA into 5′-ppp dsRNA and induces IFN-β through the RIG-I and MDA-5 pathways. Inactivation of the SFV replicase RdRp activity prevents IFN-β induction. These IFN-inducing modified host cell RNAs are abundantly produced during both wild-type SFV and its non-pathogenic mutant infection. Furthermore, in contrast to the wild-type SFV replicase a non-pathogenic mutant replicase triggers increased IFN-β production, which leads to a shutdown of virus replication. These results suggest that host cells can restrict RNA virus replication by detecting the products of unspecific viral replicase RdRp activity.

## Author Summary

Type I interferons (IFN) are critical for mounting effective antiviral responses by the host cells. For RNA viruses, it is believed that IFN is triggered exclusively by viral double-stranded RNA (dsRNA) or RNA containing a 5′-triphosphate (5′-ppp) that is produced during viral genome replication or transcription driven by viral replicases. Here, we provide strong evidence suggesting that the viral replicase also generates 5′-ppp dsRNA using cellular RNA templates, which trigger IFN. This finding indicates that viral replicase is capable of activating the host innate immune response, deviating from the paradigm that viral nucleic acid replication or transcription must be initiated in the host cell to trigger IFN production. Using Semliki Forest virus (SFV) as a model, we show that the magnitude of innate immune response activation by the viral replicase plays a decisive role in establishing viral infection. We demonstrate that in contrast to the wild-type SFV replicase, a non-pathogenic mutant replicase triggers increased IFN production, which leads to a shutdown of virus replication. Consequently, excessive IFN induction by the viral replicase can be dangerous for an RNA virus. Thus, we delineate a novel mechanism by which an RNA virus triggers the host cell immune response leading to RNA virus replication shutdown.

## Introduction

The innate immune system is an ancient set of host defense mechanisms that utilize germline-encoded receptors for the recognition of pathogens [1]. This set of receptors, termed pathogen recognition receptors (PRRs), binds to the pathogen's own structural or pathogen-induced molecules and triggers an anti-pathogenic cellular state through various signal transduction pathways. The set of molecules brought into the cells or induced by pathogens are called pathogen-associated molecular patterns (PAMPs) [2]. The number of different germline-encoded PRRs is limited; therefore, PAMPs represent unique structural signatures that are characteristic of several groups of pathogens [1].

In the case of RNA viruses, double-stranded RNA (dsRNA) and 5′-triphosphate (5′-ppp) RNA are the most common pathogen-characteristic molecular structures recognized by PRRs. Viral RNA replicases generate 5′-ppp RNA and/or dsRNA in ample amounts during replication and transcription of viral RNA genomes. The presence of viral dsRNA in an animal cell is an indication of the pathogen invasion and is recognized by the innate immune system as a non-self entity, as vertebrate genomes do not encode RNA-dependent RNA polymerase (RdRp) activity. Recognition of viral dsRNA by specific PRRs leads to the induction of type I interferons (IFN; e.g. IFN-α and IFN-β) [3], which promote an antiviral state of the cell by inducing several hundred genes expression [4]. In vertebrates, type I IFNs and several other cytokines mediate innate immune system signals that determine the type of response elicited by the adaptive immune system [2].

Currently, three PRR families have been identified as innate immune sensors involved in the detection of virus-specific components in cells: Toll-like receptors (TLRs), retinoic acid-inducible gene I (RIG-I)-like receptors (RLRs), and nucleotide oligomerization domain (NOD)-like receptors (NLRs). Only TLRs and RLRs, however, are important for type I IFN induction. RLRs are the primary detectors of cytosolic 5′-ppp RNA and dsRNA generated by RNA viruses [3]. In addition to dsRNA [5], host PRRs detect dsRNA with 5′-ppp ends [6], single-stranded RNA (ssRNA) [7], and viral genomic DNA [8], [9]. Thus, type I IFN production is almost exclusively triggered by the recognition of viral nucleic acids. In fact, there seem to be only two exceptions. First, TLR4 receptors present on macrophages trigger type I IFN induction in response to lipopolysaccharide, which is not nucleic acid [10]. Second, TLR2 receptors present on “inflammatory” monocytes were recently reported to activate type I IFN in response to as yet unidentified components of DNA viruses [11]. For RNA viruses, however, it is believed that type I IFN is triggered exclusively by viral dsRNA [5] or 5′-ppp dsRNA [6], [12]. Accordingly, the presence of viral nucleic acids in a host cell is the absolute requirement for RNA virus detection and type I IFN production.

The main function for the viral RNA replicase is to drive the replication and transcription of viral RNA. Recently, however, several observations regarding an unusual extra property of positive-strand RNA virus replicases have been reported. In particular, the transient expression of the HCV replicase was shown to activate the IFN-β promoter in several human cell lines [13], [14], and IFN-β promoter activation was also observed for the human norovirus replicase [15]. Moreover, transgenic mice expressing the replicase of Theiler's murine encephalitis virus (TMEV) were resistant to infection by this virus and showed increased basal IFN levels [16]. Therefore, the expression of the viral replicase in the absence of a replication-competent viral genome can activate the IFN-β promoter. However, the mechanism and role of this innate immune response activation on the viral life cycle have not been determined.

In this report, we use Semliki Forest virus (SFV, *Alphavirus*) as a model to study the innate immune response of host cells to infection by a positive-strand RNA virus. Alphaviruses are single-stranded RNA (ssRNA) viruses that replicate in the cytoplasm. SFV RNA is translated into a replicase polyprotein, which consists of four multifunctional, non-structural viral proteins (nsP1, nsP2, nsP3, and nsP4). The replicase polyprotein is remodeled by the nsP2 protease through sequential cleavages to produce replicases with different specificities [17]. The core RdRp of the SFV replicase is represented by nsP4, which contains a conserved catalytic GDD triad [18]. However, when nsP4 is expressed separately from the other replicase proteins, it cannot function as an RdRp [19]. It has been reported that the alphavirus nsP2 protein is absolutely required for the suppression of the host cell antiviral response either by inducing macromolecular synthesis shutoff or by targeting the catalytic subunit of the cellular RNA polymerase [20], [21], [22]. In addition, it was demonstrated that nsP2 expression efficiently inhibited IFN-induced JAK-STAT signalling indicating a shutoff-independent mechanism [23]. However, the infection of heterogeneous bone-marrow-derived dendritic cells (BMDC) by wild-type Sindbis virus (SIN) resulted in high IFN-β induction, no prominent shutoff, and self-limiting infection [24]. In addition, IFN-α/β is very potently induced within the first 12 hr post-infection in the serum of mice infected by wild type SIN [25]. Thus, a simple model in which nsP2 antagonizes IFN-α/β and promotes virus infection cannot fully explain the virus phenotypes in non-established cell lines and animals [26]. On the other hand, SFV replication is associated with the production of viral dsRNA replication intermediates that are thought to be the molecules used by the host to detect SFV infection, even though these intermediates are located inside of the membrane-bound replicase complexes. Therefore, similar to other viruses, SFV possesses mechanism(s) that result in the prevention and/or suppression of the antiviral IFN response. A non-pathogenic SFV4 mutant that contains a mutation that disrupts the nuclear localization sequence (NLS) of nsP2 (SFV4-RDR) has been reported to be deficient in suppressing the antiviral IFN response [27], [28], [29], [30].

Here, we present evidence for a novel mechanism by which mouse embryonic fibroblasts (MEFs) detect SFV infection. This detection results in IFN-β induction and, in the case of MEF infection with a non-pathogenic SFV4-RDR, the shutdown of virus replication. The reconstitution of SFV replication by uncoupling the wild-type and mutant replicase expression from the viral RNA template led to the identification of the viral replicase as the enzyme responsible for IFN-β induction. Remarkably, wild-type and mutant SFV replicases were capable of IFN-β induction in the absence of viral RNA replication or transcription. IFN-β induction resulted from the production of IFN-inducing RNA ligands generated from cellular RNAs that could activate RIG-I and MDA-5. Increased levels of IFN-β were induced upon the expression of the viral replicase with a mutation in the NLS of nsP2, while inactivation of RdRp activity of nsP4 blocked the induction of IFN-β. These results indicate that during SFV infection, two concurrent processes are driven by the viral replicase. First, the viral replicase drives the replication and transcription of the viral genome. Second, the viral replicase generates PAMPs using host cell RNA as a template. Thus, the innate immune recognition of RNA virus infection is more complex than pattern recognition, which is based on the detection of invariant structures present in pathogens or viral replication intermediates. Furthermore, viruses must find ways to circumvent the increased efficiency of their recognition by utilizing powerful mechanisms targeting innate immune response. A failure to interfere with host cell detection mechanisms results in virus replication shutdown and elimination.

## Results

### SFV Replicase Triggers Induction of Type I IFN

All previous studies on IFN induction by alphaviruses have been performed using either viral infection or transfection of viral replicons. It has been shown that wild-type SFV4 is capable of IFN-β induction upon infection of a host cell [27], [31], [32]. It is not clear whether this is valid for all alphaviruses as no type I IFN induction was observed in MEFs infected with wild type SIN [33]. In contrast it had been shown that transfection of cells with a SIN replicon, a modified alphavirus genome that replicates but does not produce virus due to the replacement of the viral structural genes with a heterologous sequences, also triggers IFN-β production [34]. Introduction of an RR^649^R→RD^649^R mutation into the NLS of SFV4 nsP2 resulted in a virus, termed SFV4-RDR, that induced almost seven times more IFN-β than the wild-type virus [27]. Hence, the replicase region is essential for triggering IFN-β induction and also for limiting that response. However, the exact mechanism(s) by which IFN-β is triggered during alphavirus replication and the reasons why the mutant virus induces more IFN production than the wild-type virus are poorly understood.

**Figure 1:**
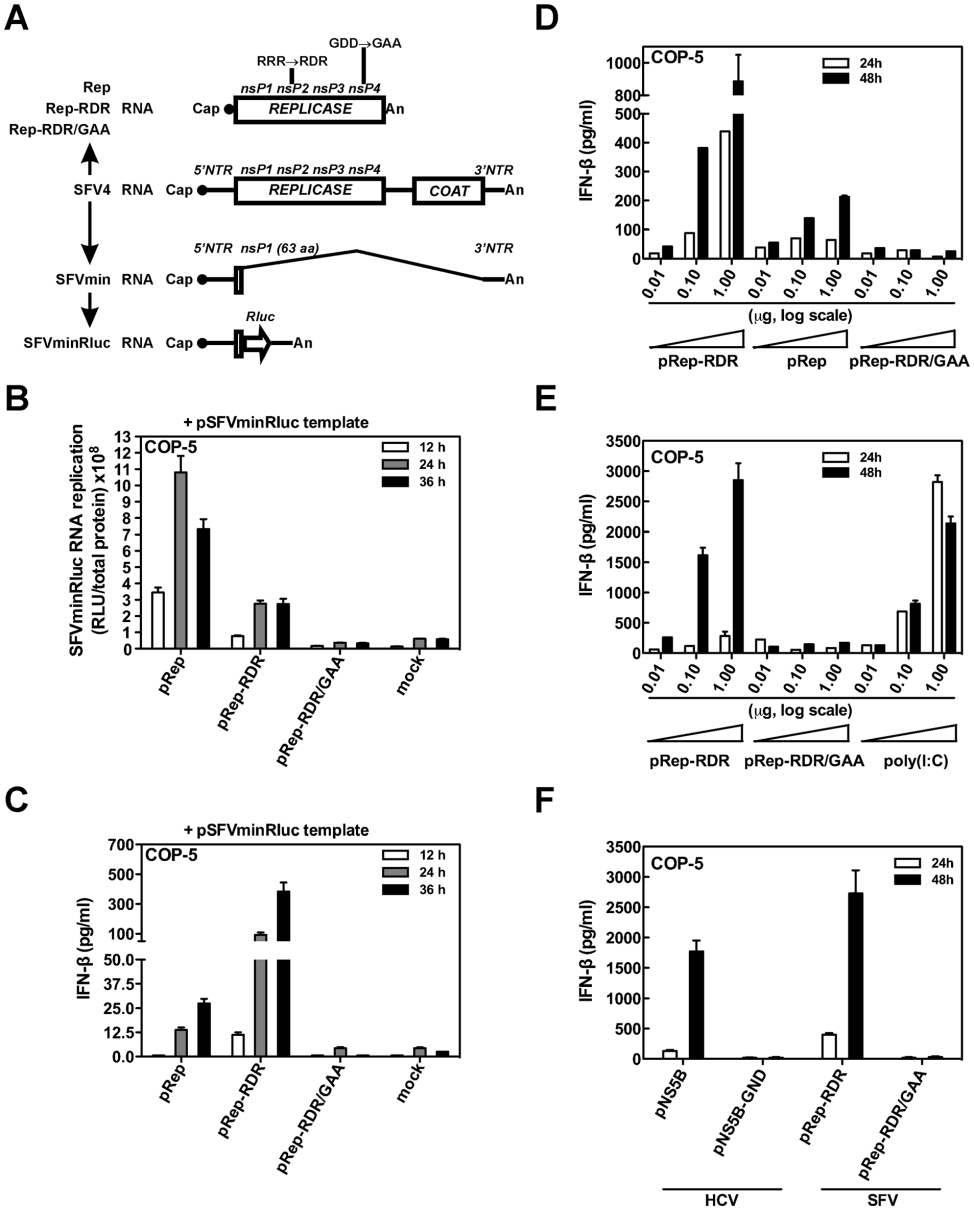
Transient expression of SFV replicase with intact RdRp activity is sufficient for IFN-β induction. (A) Scheme for the SFV replication reconstitution system. Capped RNAs directing the translation of the SFV replicase variants (Rep, Rep-RDR, and Rep-RDR/GAA) were expressed from the corresponding plasmid DNAs. Capped SFVminRluc RNA was derived from the SFV4 RNA by replacing the viral coding sequences, with the exception of the N-terminal 63 aa of nsP1, with the *Rluc* reporter coding sequence. NTR, non-translated region. (B and C) COP-5 cells were transfected with a pSFVminRluc in combination with pRep, pRep-RDR, pRep-RDR/GAA or an irrelevant “carrier” DNA (mock). At the indicated times, cell lysates were prepared for the Renilla luciferase reporter assay (B), and IFN-β secreted into the cell culture supernatants (C) was measured by ELISA. (D) COP-5 cells were transfected with different amounts of pRep, pRep-RDR, or pRep-GAA to measure IFN-β production as described in (C). (E) COP-5 cells were transfected with different amounts of pRep-RDR, pRep-RDR/GAA or poly(I:C) dsRNA, and IFN-β was measured as described in (C). The total amount of DNA in the transfections (D and E) was kept constant using “stuffer” plasmid, which encoded the EGFP. (F) COP-5 cells were transfected with equal amounts (1 µg) of pRep-RDR, pRep-RDR/GAA, pNS5B, or pNS5B-GND, and IFN-β was measured as described in (C). Error bars represent the standard deviation of two (B–D) and three (E and F) experiments.

We have previously demonstrated that SFV replicase that was expressed from a non-replicating mRNA specifically and efficiently replicated the SFV RNA template when the template was provided as a separate RNA molecule [35], and this finding was later confirmed [36], [37]. Therefore, the approach used in the current study was based on the uncoupling of replicase expression from the viral RNA template. For this purpose, codon-optimized DNA sequences encoding only the replicases of SFV4 and SFV4-RDR were inserted under the control of a heterologous promoter to produce the pRep and pRep-RDR plasmids (Figure 1A). As a control, we generated a pRep-RDR/GAA plasmid containing two point mutations in the RdRp-specific catalytic motif [18] of the nsP4 protein (GD^466^D^467^→GA^466^A^467^) that inactivate the RdRp activity of the viral replicase. For accurate reconstitution of viral replication, we also produced the pSFVminRluc plasmid encoding a minimal viral RNA template, which contained the coding sequence of the Renilla luciferase (Rluc) reporter flanked by cis-sequences that are required for efficient viral replication (Figure 1A). To determine the basal level of Rluc activity expressed from the RNA template, we transfected mouse fibroblast COP-5 cells [38] with pSFVminRluc and compared the results to those obtained with co-transfection of COP-5 cells with pSFVminRluc and either pRep or pRep-RDR. As expected, the presence of functional replicase resulted in the accumulation of intracellular Rluc activity that exceeded the basal level obtained with the template alone. In contrast, no accumulation of Rluc activity above basal level was observed when COP-5 cells were co-transfected with pSFVminRluc and pRep-RDR/GAA (Figure 1B). Consequently, the intact RdRp activity of the viral replicase was responsible for the amplification of Rluc activity. This clearly indicated that replication of the SFVminRluc RNA template was driven by the viral replicases expressed from the pRep and pRep-RDR plasmids. Although the replication of SFVminRluc by the wild-type SFV replicase was more efficient (Figure 1B), we found that pSFVminRluc co-transfection with pRep-RDR induced an increase in IFN-β production that was an order of magnitude greater that that induced by co-transfection with pRep (Figure 1C).

There are at least three potential mechanisms by which pSFVminRluc co-transfection with pRep-RDR could lead to enhanced IFN-β induction. First, viral RNA replication products generated by Rep-RDR may be more abundant and/or more accessible to the host cell innate immune sensors than the viral RNA replication products generated by wild-type Rep. Second, the wild-type replicase of SFV may interfere with IFN-β production and/or signaling by multiple mechanisms [20], [21], [27], [28]. Alternatively, Rep-RDR may have additional, non-viral RNA template targets in the host cell transcriptome. To determine the exact mechanism, we transfected mouse COP-5 fibroblast cells with pRep, pRep-RDR, and pRep-GAA and measured the amount of IFN-β in the cell culture medium at different time points. Unexpectedly, the expression of the SFV replicase induced robust IFN-β secretion by the transfected cells. Rep-RDR induced approximately 3–4 times more IFN-β production than Rep, whereas Rep-GAA did not induce IFN-β production (Figure 1D). The expression of the SFV replicase subunits became readily detectable in a western blot assay only when pRep, pRep-RDR, and pRep-GAA were used at the highest dose, arguing against the possibility of over-expression (Figure S1). These results indicate that the SFV replicase is capable of inducing IFN-β in the absence of replication-competent viral RNA. Thus, the induction of IFN-β during SFV infection may not only be caused by the presence of viral RNA replication or transcription intermediates but may also result from additional intrinsic properties of the functional viral replicase.

We next wanted to compare the kinetics of IFN induction in COP-5 cells transfected with either pRep-RDR or poly(I:C), which is known to induce IFN-β [5]. To achieve the comparable IFN induction we needed to increase the SFV replicase expression level, which was accomplished by the exchange of the promoter in pRep-RDR and pRep-RDR/GAA (Figure S2). Poly(I:C) transfection resulted in steady, and at the highest dose, declining levels of IFN-β, whereas the SFV replicase expression triggered a delayed but very potent accumulation of IFN-β (Figure 1E). To determine whether the expression of the SFV replicase in primary MEFs also leads to IFN-β induction, we transfected MEFs with pRep-RDR, pRep-RDR/GAA DNA, or poly(I:C) dsRNA. In the MEFs, SFV replicase expression induced IFN-β secretion, whereas Rep-RDR/GAA again failed to induce IFN-β production (Figure S3). Chloroquine treatment, which was used to avoid the TLR-9-dependent induction of IFN-β in response to DNA [39], had no effect on IFN-β induction. Therefore, transfection of pRep-RDR into primary MEFs also triggers IFN-β production. Taken together, these results demonstrate that SFV replicase expression triggers the accumulation of previously unknown PAMPs, which induce an innate immune response.

Several reports demonstrated that the transient expression of a single-subunit HCV replicase (NS5B, nonstructural protein 5B) activated the IFN-β promoter [13], [14], [40], [41]. Hence, we wanted to compare the abilities of SFV and HCV replicases to trigger the IFN-β induction. Corresponding HCV replicase plasmids encoding active NS5B (pNS5B) and the NS5B with inactivated catalytic motif (pNS5B-GND, GD^318^D→GN^318^D) were generated. Transfection of pRep-RDR and pNS5B into COP-5 cells resulted in a similar and potent IFN-β production, whereas pRep-RDR/GAA and pNS5B-GND failed to induce interferon (Figure 1F). Thus, the ability to induce IFN-β expression is a property shared by replicases of positive-strand RNA viruses belonging to at least two different virus families (*Togaviridae* and *Flaviviridae*).

### RIG-I and MDA-5 Detect Novel Products of SFV Replicase Activity

**Figure 2:**
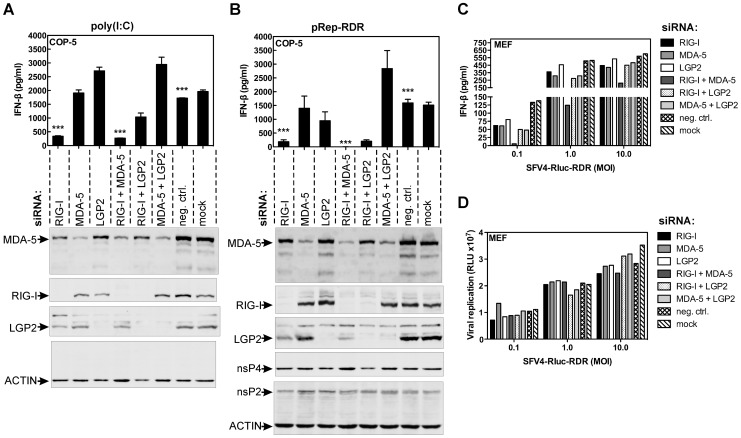
Mutant SFV Replicase triggers IFN-β in a RIG-I- and MDA-5-dependent manner. (A) COP-5 cells were transfected with siRNAs against MDA-5, RIG-I, and LGP2 or combinations thereof. After 48 hr, cells were transfected with poly(I:C) dsRNA, and after 24 hr, the amount of secreted IFN-β was measured by ELISA (upper panel). The efficiency of RLR protein knockdown was assessed by immunoblot assay (lower panel). Cells lysates were separated by SDS-PAGE and immunoblotted with different antibodies. Neg. ctrl., negative control non-targeting siRNA; mock, transfection without siRNA. (B) COP-5 cells were transfected with siRNAs as described in (A). After 48 hr, cells were transfected with pRep-RDR plasmid DNA, and after 48 hr, the amount of secreted IFN-β was measured as described in (A) (upper panel). The RLR knockdown efficiency (lower panel) was assessed as described in (A). Expression of SFV replicase was analyzed using antibodies against nsP4 and nsP2; ACTIN was used as loading control. (C and D) MEFs were transfected with siRNAs as described in (A). After 48 hr, cells were infected with SFV4-Rluc-RDR at different MOIs. After an additional 12 hr, the amount of IFN-β was measured (C) and cell lysates were prepared for the Renilla luciferase reporter assay (D). Immunoblots (A and B) and panels (C and D) are representative examples of two independent experiments. Error bars (A and B) represent the standard deviation of three experiments. ***p<0.001 (t-test).

RNA viruses can be recognized by either MDA-5, RIG-I, or a combination of the two [3]. Therefore, we tested whether these RLRs would be involved in the detection of PAMPs produced by SFV replicase RdRp activity. We transfected COP-5 cells with siRNAs targeting the mouse *Ddx58*, *Ifih1*, and *Dhx58* mRNAs, which encode the RIG-I, MDA-5, and LGP2 proteins, respectively. As a control, we used dsRNA poly(I:C) preparations shorter than ∼1.5 kbp in length, as dsRNAs of this size have been shown to induce IFN-β primarily through RIG-I [5]. As expected, silencing the expression of RIG-I either alone or in combination with MDA-5 strongly inhibited IFN-β induction by poly(I:C), which indicated that RIG-I is the primary poly(I:C) sensor in COP-5 cells (Figure 2A). Silencing both RIG-I and LGP2 expression enhanced IFN-β production as compared to silencing RIG-I alone, whereas knockdown of LGP2 expression alone or in combination with MDA-5 resulted in increased IFN-β production. These observations are consistent with the role of LGP2 as a feedback inhibitor of antiviral signaling [42] and indicate that there is both competition and interplay between receptors for a common substrate [43]. We then transfected pRep-RDR into COP-5 cells treated with siRNAs and measured IFN-β production. Again, silencing the expression of RIG-I alone strongly inhibited IFN-β induction, whereas knockdown of both RIG-I and MDA-5 blocked IFN-β production completely (Figure 2B). In this case, knockdown of RIG-I in combination with LGP2 did not enhance the IFN-β production as compared to the effect of RIG-I knockdown alone (compare Figures 2A and 2B). Notably, knockdown of LGP2 alone also did not increase IFN-β induction, whereas silencing of both MDA-5 and LGP2 did increase IFN-β induction. Importantly, silencing the expression of RLR sensors by different siRNAs gave essentially the same results, indicating that observed effects were not due to off-target effects (data not shown). The possibility that the siRNA transfections also affected the expression of the SFV replicase and thus contributed to the alteration of IFN-β production was also excluded, as the amounts of SFV nsP2 and nsP4 in different cell lysates were roughly identical (Figure 2B). Furthermore, the amounts of the replicase proteins in the transfected cells never exceeded the amounts in the cells that were infected with the corresponding virus. Thus, this analysis also excluded any possibility that the IFN-β induction was an artifact that resulted from the transient over-expression of the viral replicase. Therefore, the production of novel PAMPs is a natural function of SFV replicase RdRp activity, and RIG-I is the major sensor for these products. Moreover, for PAMPs generated by the SFV replicase, these data suggest that MDA-5 serves as an auxiliary sensor that also contributes to IFN-β induction, albeit to a much lesser extent than RIG-I.

It has been previously reported that the sensors for PAMPs generated during alphavirus infection include MDA-5 and RIG-I [31], [32]. Therefore, we determined which RLR sensors would be important for the detection of the mutant SFV4-RDR. For this purpose, SFV4-Rluc and SFV4-Rluc-RDR reporter viruses containing the *Renilla* luciferase (Rluc) coding sequence were used; in these viruses, the Rluc reporter is inserted between the coding sequences of nsP3 and nsP4, and this insertion does not interfere with correct replicase polyprotein cleavage. In addition, the expression of the reporter gene for these viruses is proportional to the RNA genome copy number [44]. Following transfection of the siRNAs, we infected MEFs with SFV4-Rluc-RDR at different multiplicity of infection (MOI) values. Analysis of the Rluc reporter activity indicated that transfection of the siRNAs had little or no effect on mutant SFV replication in MEFs (Figure 2D). In contrast, knockdown of both RIG-I and MDA-5 decreased IFN-β secretion at every MOI tested (Figure 2C). At the lowest MOI tested, it became clear that both RIG-I and MDA-5 were equally important for SFV recognition in MEFs (Figure 2C). These results indicate that the same RLR sensors are involved in both the detection of infection by the SFV and novel products of its replicase RdRp activity. However, MDA-5 knockdown had relatively little effect on the recognition of PAMPs generated by the intrinsic RdRp activity of the viral replicase, whereas the contribution of MDA-5 to the recognition of infection by the virus was similar to that of RIG-I.

### SFV Replicase Generates IFN-inducing RNA Containing Double-Stranded RNA and 5′-Triphosphate

**Figure 3:**
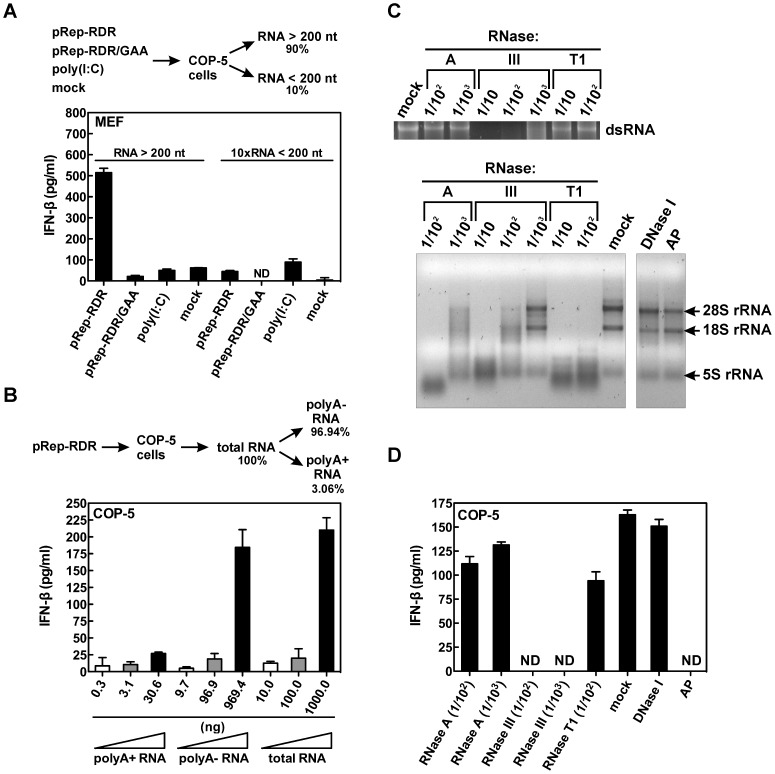
Properties of IFN-inducing RNA generated by SFV replicase. (A) COP-5 cells were transfected with either pRep-RDR, pRep-RDR/GAA or poly(I:C) or were mock-transfected. After 48 hr, the cells were lysed and the total RNA was size-fractionated on a silica column. The two resulting fractions, containing either large RNAs (>200 nt) or small RNAs (<200 nt), were transfected into MEFs, and IFN-β levels were measured by ELISA after 24 hr. Mock, transfection without DNA or RNA; ND, not detectable; 10×RNA <200, 10-fold molar excess of small RNAs as compared to large RNAs. (B) COP-5 cells transfected with pRep-RDR were lysed at 48 hr post transfection. The total RNA was extracted and fractionated into polyadenylated and non-polyadenylated RNA fractions using oligo(dT)-affinity chromatography. Increasing amounts of the obtained RNA fractions were transfected into COP-5 cells, while the total RNA amount was kept constant with “stuffer” RNA (naïve COP-5 total RNA). At 24 hr after transfection, the amount of IFN-β was determined in the cell culture medium by ELISA. polyA+, polyadenylated; polyA−, non-polyadenylated. (C) Upper panel: dsRNA probes were treated with various amounts of the indicated RNase, separated on agarose gel, and then visualized by ethidium bromide staining. Lower panel: Total RNA was extracted from pRep-RDR-transfected cells and digested with various amounts of the indicated RNase or treated with DNase I or alkaline phosphatase (AP) and analyzed as described in (C, upper panel). Mock, no enzyme added. (D) RNAs (C, lower panel) were transfected into COP-5 cells, and ELISA was used to measure the amount of secreted IFN-β after 24 hr. Error bars (A, B, and D) represent the standard deviation of two experiments.

Given that SFV replicase RdRp activity was absolutely necessary for the generation of PAMP structures to trigger the RIG-I pathway and IFN-β induction, we expected that these PAMPs may in fact be RNAs. Furthermore, the dominant role of RIG-I in the recognition of these PAMPs suggests that these potential RNAs must be shorter than the full-length dsRNA replication intermediates produced during viral infection. To address this question regarding the nature of these PAMPs, we isolated total RNA from COP-5 cells transfected with pRep-RDR, pRep-RDR/GAA and poly(I:C) and then separated the large RNAs fraction (>200 nt) from the fraction containing smaller RNAs (<200 nt). Only the large RNAs from COP-5 cells transfected with pRep-RDR were capable of inducing IFN-β in MEFs, whereas large RNA fraction from either pRep-RDR/GAA- or poly(I:C)-transfected cells was incapable of inducing a comparable IFN response (Figure 3A). Consequently, the RNA extracted from poly(I:C)-transfected cells, which was used for the secondary MEFs transfection, contained very little of the originally-transfected poly(I:C). Therefore, this RNA was unable to trigger the RIG-I pathway following re-transfection. Furthermore, this also indicates that the contribution of the IFN-β, translated directly from corresponding mRNAs purified from initially transfected cells, to the IFN-β production in re-transfected cells is negligible. Even when transfected at a 10-fold molar excess as compared to the large RNA fractions, the small RNA fractions were unable to trigger comparable IFN-β activity. This observation also indicates that the antiviral endoribonuclease L (RNase L), which has been reported to cleave host cell RNA to produce small RNA molecules (less than 200 nt) capable of triggering the RIG-I pathway [45], is not involved in the detection of products of SFV replicase RdRp activity.

Next, we transfected COP-5 cells with pRep-RDR and fractionated the total RNA into polyadenylated (polyA+) and nonpolyadenylated (polyA−) RNA using oligo(dT)-affinity chromatography. The polyadenylated RNAs comprised approximately 3% of the total RNA extracted from the COP-5 cells. We then transfected naïve COP-5 cells with polyA+, polyA−, or total RNA in stoichiometric amounts (polyA+ RNA : polyA− RNA : total RNA = 1 : 32 : 33) and measured the IFN-β response (Figure 3B). Approximately 90% of the IFN-β signal was induced by the nonpolyadenylated RNA, suggesting that this fraction contained the majority of the RNAs (PAMPs) generated by the SFV replicase RdRp activity.

It has been shown that RIG-I recognizes dsRNA or dsRNA containing a 5′-ppp [5], [6]. To determine the structural features of the IFN-β-inducing RNA generated by the SFV replicase, we treated RNA isolated from COP-5 cells with various ribonucleases (RNases). At the concentrations used, RNase A did not digest model dsRNA, whereas ssRNA was efficiently degraded (Figure 3C, upper and lower panels). RNase T1 is exclusively ssRNA-specific and does not degrade dsRNA, whereas RNase III should specifically digest only dsRNA unless used at a high concentration [46], which we confirmed to be the case (Figure 3C, compare upper and lower panels).

When RNA extracted from the COP-5 cells transfected with pRep-RDR was treated with RNase III, this RNA lost the ability to induce IFN-β production (Figure 3D). In contrast, RNase A had virtually no effect on the IFN-inducing activity of the RNA. Similarly, RNase T1 treatment of the RNA did not substantially alter its ability to induce IFN-β production (Figure 3D). These results suggest that the IFN-inducing RNA extracted from SFV replicase-transfected cells is in the form of dsRNA. As expected, DNase I treatment did not have any effect on the IFN-inducing of activity of the RNA. Finally, when we treated the RNA with alkaline phosphatase, the ability of the RNA to induce IFN-β was destroyed, which indicated that the terminal phosphate structure was absolutely required for IFN-β induction. Taken together, these results suggest that the SFV replicase generates ligands for RIG-I that consist of non-polyadenylated RNA species larger than 200 nt containing dsRNA regions and a terminal 5′-phosphate, which is most likely a 5′-triphosphate.

### Lysosomes and Endosomes Are Enriched in IFN-Inducing RNAs, Produced by the Viral Replicase

**Figure 4:**
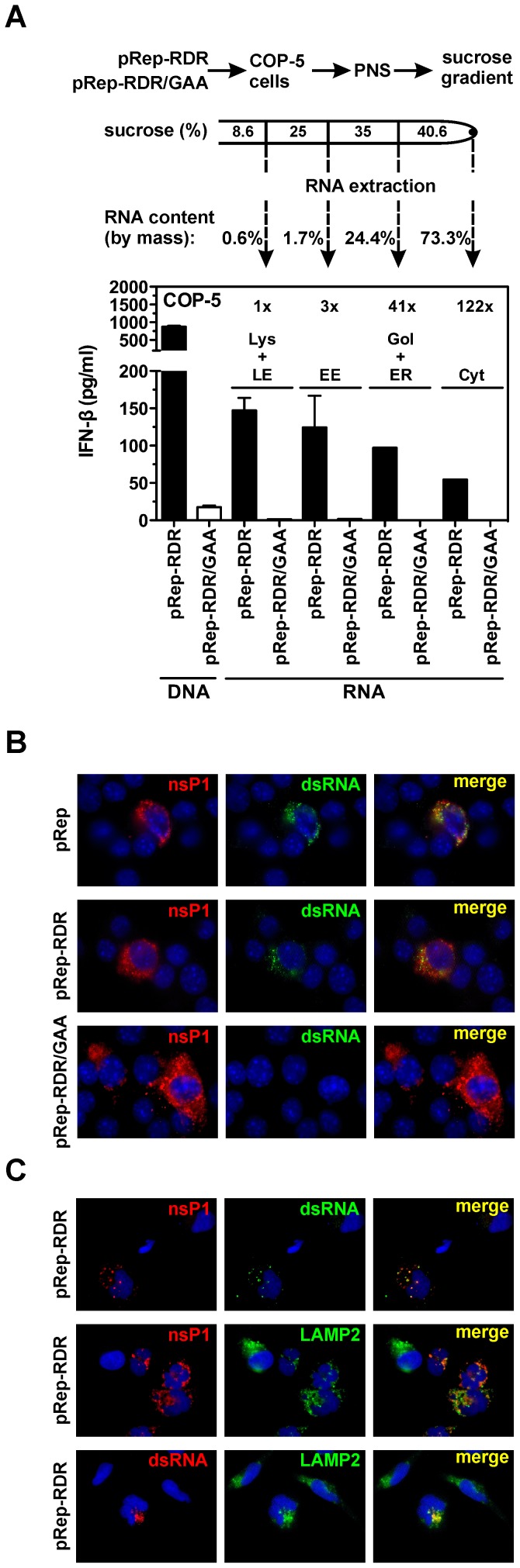
Lysosomes and endosomes are enriched in IFN-inducing RNA generated by mutant viral replicase. (A) COP-5 cells were transfected with pRep-RDR or pRep-RDR/GAA plasmid DNA. After 48 hr, post-nuclear supernatant (PNS) was prepared and fractionated by flotation in a sucrose step gradient. Subsequently, naïve COP-5 cells were transfected in equimolar amounts with RNAs extracted from each fraction, and IFN-β levels were measured by ELISA. Lys+LE, lysosomes and late endosomes; EE, early endosomes; Gol+ER, Golgi complex and endoplasmic reticulum; Cyt, cytosol. (B) COP-5 cells were transfected with pRep, pRep-RDR, or pRep-RDR/GAA plasmid DNA. After 48 hr, the cells were fixed, permeabilized and stained with anti-nsP1 and anti-dsRNA antibodies. The cells nuclei were counterstained with 4,6-diamino-2-phenylindoldihydrochloride. (C) RD cells were transfected with pRep-RDR plasmid DNA. After 48 hr, the cells were fixed, permeabilized and stained with different combinations of anti-nsP1, anti-dsRNA, and anti-LAMP2 antibodies. The nuclei were counterstained as described in (B). Error bars (A) represent the standard deviation of two experiments. All images (B and C) are representative of three independent experiments.

It has been shown that although the dsRNA containing SFV replicase complexes (spherules) are initially formed at the plasma-membrane, they are subsequently internalized [47] and localize to the cytoplasmic surface of both endosomes and lysosomes [48]. Moreover, expression of the viral replicase alone in type I IFN-deficient BHK (baby hamster kidney) cells resulted in the characteristic endo- and lysosomal localization of nsPs, although there was no spherule formation [37]. To determine whether the IFN-inducing RNAs produced by the SFV replicase associate with either endosomes or lysosomes, we performed subcellular fractionation of COP-5 cells transfected with pRep-RDR and pRep-RDR/GAA. The subcellular fractions containing various organelles were prepared from post-nuclear supernatants by flotation in sucrose step gradients. Subsequently, we extracted RNA from each of the fractions, and transfected these RNAs into naïve COP-5 cells. The RNAs extracted from each subcellular fraction of the pRep-RDR-transfected cells were capable of inducing IFN-β production; however, RNAs extracted from the endosomes and lysosomes were the most potent IFN-β inducers (Figure 4A).

Next, we determined whether the replicase-generated IFN-inducing dsRNA could be detected in transfected cells by immunofluorescence microscopy. For this purpose, we utilized the J2 monoclonal antibody, which recognizes dsRNA regions longer than ∼40 bp in length [49]. In this experiment, dsRNA-specific staining was detected in both pRep- and pRep-RDR-transfected COP-5 cells, whereas pRep-RDR/GAA-transfected cells contained no detectable dsRNA. Moreover, co-localization of dsRNA and the nsP1 of SFV was clearly detected (Figure 4B). Due to the enriched IFN-inducing RNA in endosomes and lysosomes, we further analyzed the potential co-localization between SFV nsP1 and dsRNA with the lysosomal marker protein LAMP2. The assay was performed using human rhabdomyosarcoma (RD) cells, as several mouse-specific LAMP2 antibodies produced a high background signal in COP-5 cells. Both dsRNA and LAMP2 showed co-staining with nsP1, and dsRNA and LAMP2 also showed co-staining with each other (Figure 4C). These results confirmed that the SFV replicase generates dsRNA structures and demonstrated that the dsRNA duplex region(s) is longer than ∼40 bp in length. Additionally, the endosomes and lysosomes are enriched in these IFN-inducing RNAs and consequently serve as the sites of SFV replicase docking and dsRNA generation. These findings indicate that our experimental system accurately reconstituted the conditions observed during actual SFV infection [37], [48].

### Increased Type I IFN Induction Triggers the Shutdown of Mutant SFV Replication

Infection with a virulent strain of SFV, SFV4 [50], is cytotoxic for vertebrate cells and leads to the shutdown of cellular transcription and translation. This process has, at least in part, been attributed to the properties of nsP2 [22], [51]. Approximately half of the nsP2 produced is transported to the nuclei of SFV4-infected cells [29], and disruption of the nsP2 NLS by the RR^649^R→RD^649^R mutation attenuates the pathogenicity of the corresponding virus [30]. Previous studies with SFV4 and SFV4-RDR in cells deficient for the type I IFN response showed that both viruses grew to high titers with similar kinetics [27], [30]. However, in cells with an intact type I IFN response, the kinetics of viral accumulation differed; although both viruses grew to high titers by 12 hr post-infection (h.p.i.), the SFV4-RDR titer failed to increase further, whereas the SFV4 titer continued to increase until 24 h.p.i. [27]. These findings indicate that replication of the mutant SFV4-RDR was altered in cells with intact type I IFN signaling.

**Figure 5:**
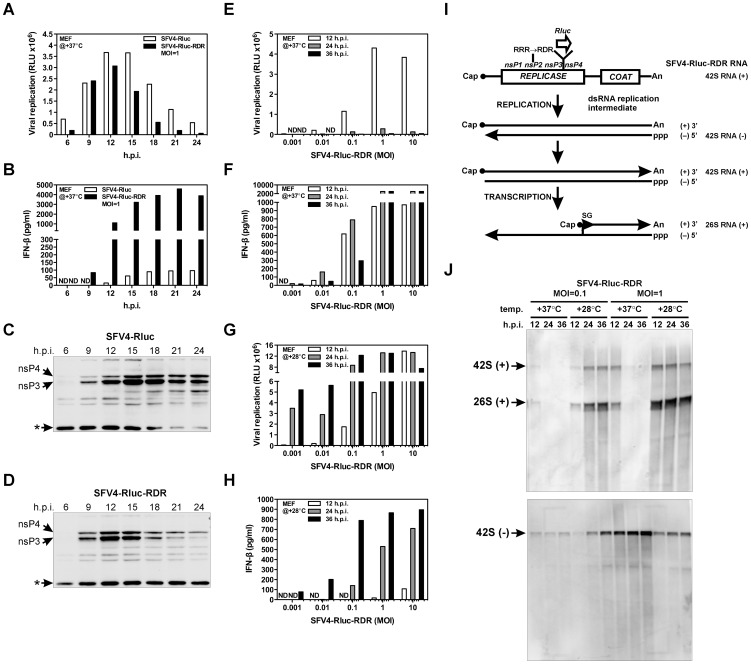
Increased type I IFN induction by viral replicase Triggers the Shutdown of Mutant SFV Replication. (A–D) MEFs were infected at 37°C with SFV4-Rluc and SFV4-Rluc-RDR at an MOI of 1. At the indicated times, cell lysates were prepared for the Renilla luciferase reporter assay (A), western blot assay (C and D), and IFN-β secreted into the cell culture supernatants (B) was measured by ELISA. RLU, relative light units; ND, not detectable; h.p.i., hr post-infection; *, non-specific cellular protein, recognized by nsP4 antibody. (E–H) MEFs were infected at 37°C (E and F) or 28°C (G and H) with the mutant SFV4-Rluc-RDR reporter virus at various MOI values prior to the performance of functional assays, as described in (A–D). (I) Scheme for capped SFV4-Rluc-RDR viral RNA genome replication and transcription driven by the viral replicase. RRR→RDR, nsP2 NLS mutation; SG, subgenomic promoter; n, variable number of residues; (+), positive-strand RNA; (−), negative-strand RNA. (J) MEFs were infected at 37°C or 28°C with the SFV4-Rluc-RDR virus at an MOI of 0.1 or an MOI of 1. Total RNA was extracted and analyzed by northern blotting with a digoxigenin-UTP labeled RNA-probe complementary to the viral positive (upper panel) or negative (lower panel) RNA strands. Equal amounts of total RNA were loaded into each lane. The data are representative of two (A, B, and J) or three (E–H) independent experiments. Immunoblots (C and D) are representative examples of two independent experiments.

The analysis of the SFV4 and SFV4-RDR replication kinetics in MEFs did not reveal any differences in viral RNA accumulation up to 7 h.p.i. [27]. To analyze the replication of these viruses in greater detail, MEFs were infected with SFV4-Rluc and SFV4-Rluc-RDR at an MOI of 1, and the Rluc activity was measured. No significant difference in the replication kinetics of either reporter virus was observed (Figure 5A). Under closer examination, however, by 24 h.p.i., the replication of the mutant virus was almost entirely suppressed and only minor cytotoxic side effects were observed, whereas the decreased replication rate of the wild-type virus was associated with death. SFV4-Rluc-induced death of MEFs was also confirmed by the disappearance of abundant cellular protein that is recognized non-specifically by the nsP4 antibody (Figure 5C). Thus, the same phenotype (decrease of replication) seems to stem from a completely different origin. Similar to previously published results [27], we observed that MEFs produced considerably more IFN-β in response to infection with the mutant virus (Figure 5B). The observed difference (up to 40-fold), however, was nearly a magnitude larger than previously reported. These results suggest that the increased IFN-β production induced by SFV4-Rluc-RDR triggered an antiviral mechanism, which led to the restriction of viral replication in MEFs.

During several independent experiments, four aspects of the results were highly reproducible. First, the peak level of viral replicase expression, as indicated by the induction of Rluc reporter activity and the production of replicase subunits, was achieved faster in response to SFV4-Rluc-RDR (12 h.p.i.) than to SFV4-Rluc (15 h.p.i.); however, the levels of viral expression were roughly equal (Figures 5A,C,D). Thus, there are no major defects in replicase production and RNA replication of SFV4-Rluc-RDR. Second, for the mutant virus, IFN-β was detectable at earlier time points in comparison to the wild-type virus (Figure 5B). Third, peak levels of secreted IFN-β were obtained after peak levels of the viral replicase nsP3 and nsP4 (RdRp) subunits were established (Figures 5B,C,D). Fourth, after achieving the peak levels nsP3 and nsP4 of SFV4-Rluc remained at a relatively steady level, whereas the levels of nsP3 and nsP4 of SFV4-Rluc-RDR gradually decreased (compare Figure 5A and 5B). Taken together, these results indicate that the viral replicase that induces IFN-β production is formed faster during infection with the mutant virus. Furthermore, there is a clear correlation between replicase accumulation and IFN-β production. However, after the peak levels of IFN-β are achieved, the replicase of the mutant virus is degraded.

To test whether the increased IFN-β production that was induced by SFV4-Rluc-RDR triggered an antiviral mechanism leading to the restriction of viral replication, MEFs were infected with SFV4-Rluc-RDR at different temperatures. When MEFs were infected with SFV4-Rluc-RDR at 28°C, the IFN-β secretion was greatly delayed and clearly reduced as compared to that produced as a result of infection performed at 37°C (Figures 5H and 5F). This difference is most likely because at 28°C, the RDR mutation does not completely prevent the nuclear localization of nsP2 [51]. This delay in the kinetics of IFN-β secretion allowed for the efficient replication and spread of the mutant virus (Figure 5G). In contrast, when MEFs were infected with the mutant virus at 37°C, virus infection was restricted to the cells that were initially infected, and importantly, there was a complete suppression of viral replication at 24 and 36 h.p.i. for every MOI tested (Figure 5E). Thus, MEFs restrict the replication of SFV4-Rluc-RDR in an IFN-β-dependent manner.

To confirm the results obtained from the Rluc reporter activity analysis, we used northern hybridization approach to directly visualize the replication products. During SFV infection viral replicase first generates negative-strand RNAs (42S RNA −), which, together with the genomic RNA, forms dsRNA intermediate (Figure 5I). The replicase then utilizes the negative-strand RNA genomes to produce both positive-strand RNA genomes (42S RNA +) and subgenomic RNAs (26S RNA +). At 28°C, all RNA species produced by the SFV4-Rluc-RDR in MEFs accumulated in a time-dependent manner, indicating that there was efficient viral replication and spread of infection (Figure 5J). In contrast, at 37°C, the viral RNAs of positive polarity were detectable at 12 h.p.i. but were subsequently unable to be detected, whereas the amount of the 42S RNA negative strand remained at a steady level (Figure 5J). These results clearly demonstrate that the IFN-induced restriction of SFV4-Rluc-RDR replication is mediated by the destruction of the viral positive strands of RNA.

### Wild-type and Mutant SFVs Generate Equal Amounts of PAMPs

**Figure 6:**
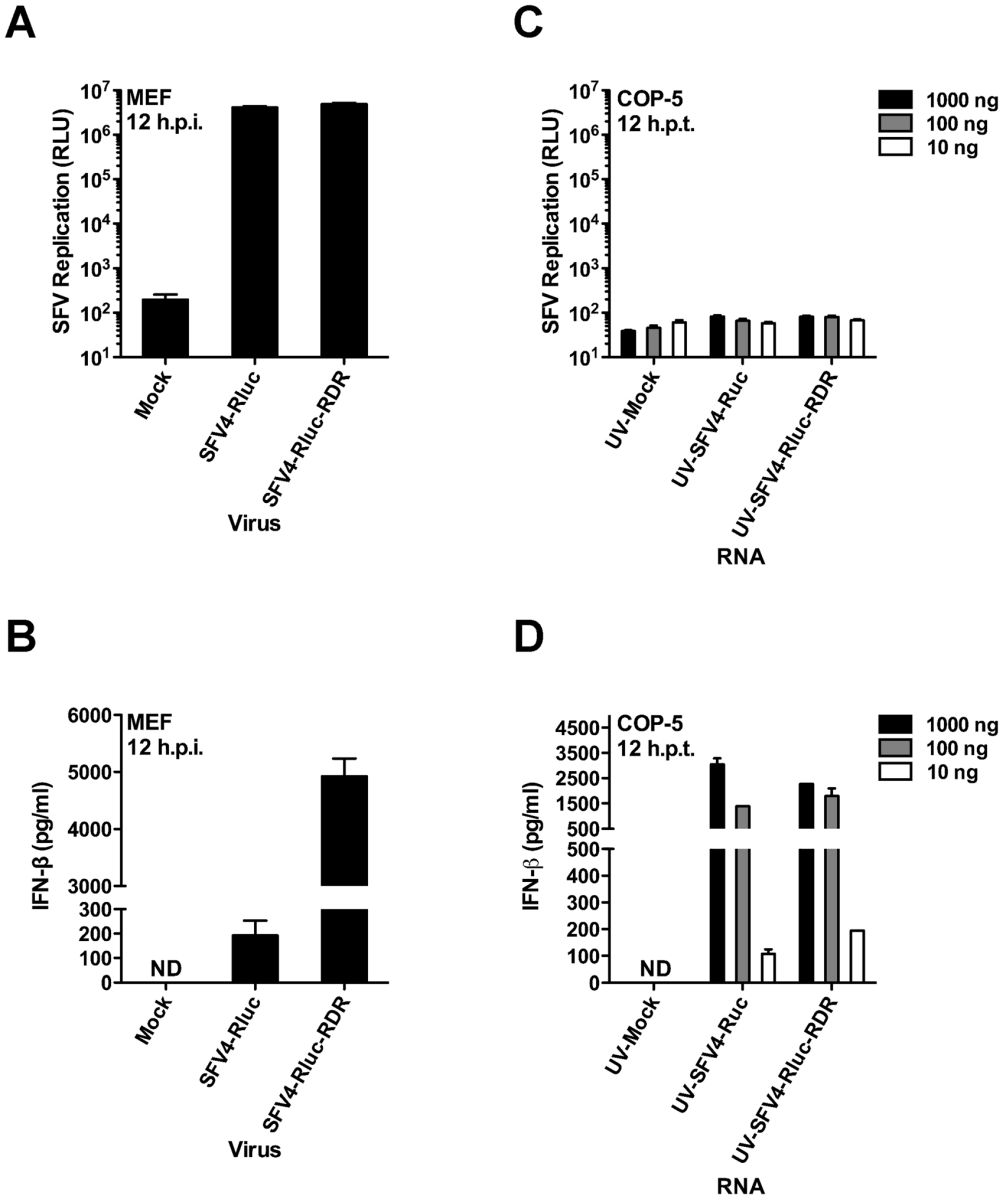
Wild-type and mutant SFVs differ in IFN induction but generate similar amounts of PAMPs. (A and B) MEFs were infected at 37°C with SFV4-Rluc and SFV4-Rluc-RDR at an MOI of 1. At 12 h.p.i., cell lysates were prepared for the Renilla luciferase reporter assay (A), and IFN-β secreted into the cell culture supernatants (B) was measured by ELISA. (C and D) Increasing amounts of Trizol reagent-extracted and UV irradiated total RNA from infected or mock-infected MEF cells were transfected into COP-5 cells, while the RNA amount was kept constant (1000 ng) with “stuffer” RNA (total RNA from naïve COP-5 cells). At 12 hr post transfection, Renilla luciferase reporter activity was measured (C), and the amount of secreted IFN-β in the cell culture medium was determined by ELISA (D). RLU, relative light units; ND, not detectable; h.p.t., hours post transfection. Error bars (A–D) represent the standard deviation of two independent experiments.

To compare the amounts of PAMP structures generated during the infection with SFV4-Rluc and SFV4-Rluc-RDR, we infected MEF cells with these viruses at a MOI of 1. The replication of each virus was analyzed at 12 h.p.i. by measuring Rluc activity. As observed in Figure 6A, both viruses replicated to the same extent, which is consistent with data from the previous experiment (Figure 5A). Also consistent with the previous data (Figure 5B), the production of IFN-β differed drastically (Figure 6B), suggesting that the wild-type virus produces less IFN-β-inducing PAMPs. To test this hypothesis, we extracted total RNA from infected MEF cells and used it to transfect COP-5 cells. To prevent the generation of additional dsRNA PAMPs after the transfection of COP-5 cells, we treated RNA samples that were extracted from the MEFs with UV (2000 µJ/cm^2^, 2 minutes). UV-treatment inactivated the infectious replication-competent SFV RNA, and as a consequence Rluc activity was fully abrogated in the RNA samples extracted from the infected cells (Figure 6C). Remarkably, the UV-treated total RNA extracted from the MEF cells infected with both viruses induced almost identical amounts of IFN-β in the COP-5 cells at all doses tested (Figure 6D). Thus, the wild-type and mutant SFV viruses generated equal amounts of PAMP structures during infection. Consequently, the wild-type virus, but not the mutant virus, efficiently blocks either the access of the RLR machinery to the PAMPs or disrupts host cell antiviral signaling (see the Discussion).

### IFN-β Induction during SFV Infection Can Not Be Exclusively Explained by the Presence of Virus-specific RNAs

Next, we wanted to estimate the relative contributions of different RNAs generated by the viral replicase during the infection to IFN-β induction. To address this question, we needed to separate the viral RNA and its replicative dsRNA form from the dsRNAs of different origin. Here, we utilized the information resulting from the pioneering works on alphavirus RNA replication, which have shown that the only species of SFV-specific RNA of negative polarity detected in infected cells were the 42S (-) RNA strands [52], [53], [54], [55]. In addition, negative strands of replicative forms of alphaviruses either contain polyU sequences that are shorter than the corresponding polyA sequence at the 3′-terminus of the 42S genomic positive strand [53] or do not contain polyU sequences at all [56], [57]. In both cases the structure of SFV replicative dsRNA is ideal for purification by oligo(dT)-affinity chromatography similarly to both genomic (42S) and subgenomic (26S) single-stranded RNAs of SFV. Thus, polyA− RNA fraction, depleted from all types of SFV RNAs but containing the large majority of novel alphavirus replicase generated PAMPs (Figure 3B), could be obtained.

**Figure 7:**
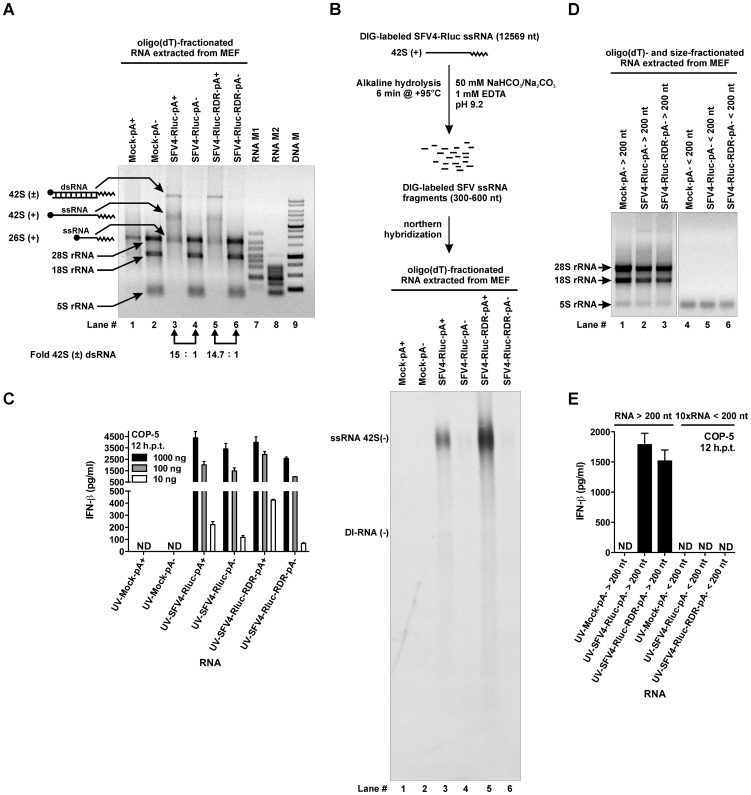
IFN-β induction during SFV infection can not be exclusively explained by the presence of virus-specific RNAs. (A) Polyadenylated (polyA+) and non-polyadenylated (polyA−) RNA fractions obtained by oligo(dT)-affinity chromatography fractionation from total RNA extracted from mock-, SFV4-Rluc-, and SFV4-Rluc-RDR-infected MEF cells. RNA samples (500 ng/lane) were resolved using electrophoresis on a non-denaturing 0.8% agarose gel and visualized by ethidium bromide staining. Quantitation of the amounts of 42S (±) dsRNA was performed with ImageJ; results are shown at the bottom of lanes 3 and 4, 5 and 6. pA+, polyadenylated RNA fraction; pA−, non-polyadenylated RNA fraction; RNA M1, RNA marker 1 (top – 6, 4, 3, 2, 1.5, 1.0, 0.5, 0.2 – bottom, Knts); RNA M2, RNA marker 2 (top – 1, 0.8, 0.6, 0.4, 0.3, 0.2, 0.1 – bottom, Knts); DNA M, DNA marker (top – 10, 8, 6, 5, 4, 3, 2.5, 2, 1.5, 1.0, 0.75, 0.5, 0.25 – bottom, Kbps). (B) PolyA+ and polyA− RNA fractions shown in (A) were resolved using electrophoresis on denaturing formaldehyde 0.8% agarose gel and transferred to nylon membrane. Subsequently, northern hybridization analysis with fragmented DIG-labeled SFV4-Rluc RNA probe (200 ng/ml) was performed. Equal amounts of RNA (30 ng) were loaded into each lane. (C) The increasing amounts of RNAs shown in (A) were UV-treated and transfected into COP-5 cells, while the total RNA amount was kept constant with “stuffer” RNA (total RNA from naïve COP-5 cells). At 12 hr post transfection, the amount of IFN-β was determined in the cell culture medium by ELISA. ND, not detectable; h.p.t., hours post transfection. (D) The non-polyadenylated RNA fractions shown in (A) were size-fractionated on silica-columns, and 1/10 of the volume of RNA was analyzed on a non-denaturing 0.8% agarose gel; RNAs were visualized with ethidium bromide staining. (E) Large RNAs (>200 nt) or small RNAs (<200 nt) shown in (D) were transfected into COP-5 cells, and IFN-β levels were measured by ELISA after 12 hr. ND, not detectable; h.p.t., hours post transfection; 10×RNA <200, 10-fold molar excess of small RNAs compared with large RNAs. Error bars (C and E) represent the standard deviation of three independent experiments.

To address this question experimentally, we fractionated total RNA extracted from mock-, SFV4-Rluc-, and SFV4-Rluc-RDR-infected MEF cells (Figure 6) using oligo(dT)-affinity chromatography. The polyA+ RNA comprised approximately 5.6%, 10.8%, and 8.1% of the total RNA extracted from the mock-, SFV4-Rluc-, and SFV4-Rluc-RDR-infected MEF cells, respectively. Consequently, SFV increases the ratio of polyA+ RNA to polyA− RNA by synthesizing its own polyadenylated positive-strand RNAs. Next, equal amounts of the RNAs (polyA+ RNA : polyA− RNA = 1∶1, mass ratio) were resolved by performing electrophoresis on a native agarose gel and visualized by ethidium bromide staining. Only three major viral RNA species were present in the fraction containing the polyA+ RNAs that were isolated from the infected MEFs: 26S (+)strand, 42S (+)strand, and 42S (±) dsRNA (Figure 7A, lanes 3 and 5). The double-stranded nature of the 42S (±) RNA was confirmed by staining with acridine orange, as well as by its absence on a denaturing agarose gel (data not shown). These viral RNA species were also present in the corresponding polyA− RNA fractions (Figure 7A, lanes 4 and 6), but in highly reduced amounts. We found that polyA+ RNA fractions contained approximately 15-fold more viral dsRNA than the respective polyA− RNA fractions (Figure 7A; lanes 3 and 4, 5 and 6). The ratio of infectious units (42S(+) RNA genomes) between the two RNA fractions obtained from SFV-Rluc infected cells was also established using an infectious center assay. The infectivity was found to be 4.3×10^5^ pfu/µg (plaque forming units per microgram) and 3×10^4^ pfu/µg for polyA+ and polyA− RNAs, respectively, thus confirming the approximately 15-fold difference. In addition, the high infectivity of the isolated RNA reflected the high quality of the RNA and lack of RNA degradation during purification and fractionation.

The (+) strands of SFV RNAs have 5′ cap structure and, on their own, cannot serve as PAMPs. In contrast, the (−) strands of SFV genome RNA or the (−) strands of defective interfering RNA (DI-RNA) can be efficient PAMPs since they possess 5′-ppp structure. Therefore, we wanted to analyze which types of viral RNA (−) strands were present in both fractions of RNA. For this purpose, equal amounts of the RNAs (polyA+ RNA : polyA− RNA = 1∶1, mass ratio) were resolved by performing electrophoresis on a denaturing formaldehyde agarose gel and transferred to a nylon membrane. Next, we shredded the labeled full-length positive strand RNA of SFV4-Rluc into pieces with length of 300–600 nucleotides and used the obtained mixture as probe for northern hybridization analysis (Figure 7B). Only one major RNA species, corresponding to 42S(−) viral RNA, were detected in these experiments. The absence of additional major RNA species on the northern blot indicated that there were no incomplete or fragmented 42S (−) strands in cells infected with either SFV4-Rluc or SFV4-Rluc-RDR (Figure 7B). However, a single discrete negative-stand RNA present in trace amounts was detected in the polyA+ fractions of infected cells; its smaller size suggested that it could represent the negative strand of DI-RNA. Importantly, northern hybridization analysis revealed that the viral RNAs of negative polarity were present in abundance in polyA+ fractions and were barely detectable in polyA− fractions. For the RNA isolated from SFV4-Rluc infected cells, we found that polyA+ RNA fraction contained ∼15-fold more viral 42S(−) RNA than polyA− RNA fraction; the difference for RNAs obtained from SFV4-Rluc-RDR infected cells was even more prominent (Figure 7B). Moreover, for SFV4-Rluc infected cells, the nearly identical 15-fold excess of viral 42S (±) dsRNA and 42S (−) RNA species in the polyA+ RNA fraction strongly suggested that the latter species were derived from the former and no free 42S (−) strands existed in infected cells. Collectively, performed experiments demonstrated that the viral ssRNAs and dsRNAs fractionated with a similar efficiency and were present in 15-fold excess in the corresponding polyA+ RNA fractions.

Subsequently, we wanted to compare the potential of polyA+ and polyA− RNA fractions isolated from infected cells to induce the IFN-β. To block the possibility of additional PAMPs generation from infectious RNA, the polyA+ and polyA− RNA fractions were UV-treated as described above before being used in subsequent assays. The transfection experiments revealed that the virus-induced PAMPs were potent inducers of IFN-β expression, and at a dose of 1000 ng, both RNA fractions saturated the ability of the host cell to produce IFN-β. The same is true for 100 ng of polyA+ RNA from SFV4-Rluc-RDR infected cells. In contrast, an almost linear response of IFN-β production was observed for 100 ng and 10 ng of RNAs from SFV4-Rluc infected cells (Figure 7C). Therefore, the abilities of these RNAs to induce IFN-β expression were compared using these two RNA quantities. Based on the results of the quantification, the polyA+ RNA contains 15-fold more viral RNA than equal amounts of the corresponding polyA− RNA. Hence, if the viral dsRNA and/or negative strand RNA are the only PAMPs that are generated in the course of infection, then the transfection with 10 ng of polyA+ RNA should induce approximately 15-fold more IFN-β compared with the transfection with 10 ng of the corresponding polyA− RNA. However, as is evident from the graph in Figure 7C, this was not the case. Instead, the transfection of COP-5 cells with polyA+ and polyA− RNA fractions from SFV4-Rluc infected cells resulted only in approximately a 2-fold higher IFN-β induction in response to the polyA+ RNA species compared with the polyA− RNA. For the RNAs that were obtained from MEF cells infected with SFV4-Rluc-RDR, transfection with 10 ng of polyA+ RNA induced approximately 6.5-fold more IFN-β compared with the polyA− RNA fraction, which again is less than the values deduced from the content of viral dsRNA.

In addition to the major viral 42S (−) RNA species, a minor barely detectable and approximately six times faster migrating RNA species were observed in the polyA+ RNA fractions isolated from cells infected with SFV4-Rluc and SFV4-Rluc-RDR (Figure 7B). It has been reported that during SFV infection DI-RNA species might be generated [58], [59]. DI-RNA are viral deletion mutants that contain the RNA sequences important for replication but are unable to self-replicate and rely on virus replication machinery, similarly to our SFVminRluc RNA template [58]. The low abundance of this RNA and especially its enrichment in polyA+ fraction excludes possibility that this molecule may have significant role in IFN-β induction by polyA− RNA fraction. Nevertheless, the presence of DI-RNAs was subsequently analyzed using a highly sensitive strand-specific reverse transcription reaction on denatured RNA followed by PCR. This method allowed for the detection of negative strands of the DI-RNAs in all of the RNA samples from infected MEF cells; the length of the DI-RNA was between 1.5 and 2 kb (Figure S4). However, coherent with the results of northern blot analysis (Figure 7B) negative strands of DI-RNA were more abundantly present in polyA+ fraction. Furthermore, positive strands of DI-RNA were detected only in the polyA+ RNA fractions (Figure S4). Taken together these results confirm that DI-RNAs were efficiently removed from the polyA− RNA samples by oligo(dT) affinity chromatography and therefore could not contribute to IFN-β induction in response to this particular RNA fraction.

Next, we wanted to address whether the RNA PAMPs present in the polyA− RNA fraction from the infected cells were similar to the PAMPs generated by transfection of pRep-RDR. Most importantly, it was essential to determine if this fraction contained RNA molecules that were generated by the degradation of viral dsRNA by either RNase L or some other mechanism and would therefore lack a polyA sequence. We subfractionated the polyA− RNA fractions according to RNA size on silica-columns (Figure 7D) and transfected the fractions into COP-5 cells as described above. Only the fraction containing large (>200 nt) RNAs induced IFN-β production, whereas smaller RNAs (<200 nt), even when used in a 10-fold molar excess, failed to do so (Figure 7E). Consequently, the IFN-β that was induced by the polyA− RNAs isolated from the infected MEF cells was not dependent on RNase L and, importantly, could not be attributed to the presence of degradation products from the viral dsRNA, which could not be detected by the northern hybridization analysis due to their small size (Figure 7B). Taken together, these results demonstrate that polyA− RNAs isolated from SFV infected MEF cells contain large amounts of potent IFN-β inducers. These inducers are not viral RNAs and therefore must have been produced by the viral replicase using host cell RNAs as templates as it was observed in case of cells transfected with pRep.

### SFV Replicase Transcribes Host Cell RNA, Triggering IFN-β Induction during SFV Infection

**Figure 8:**
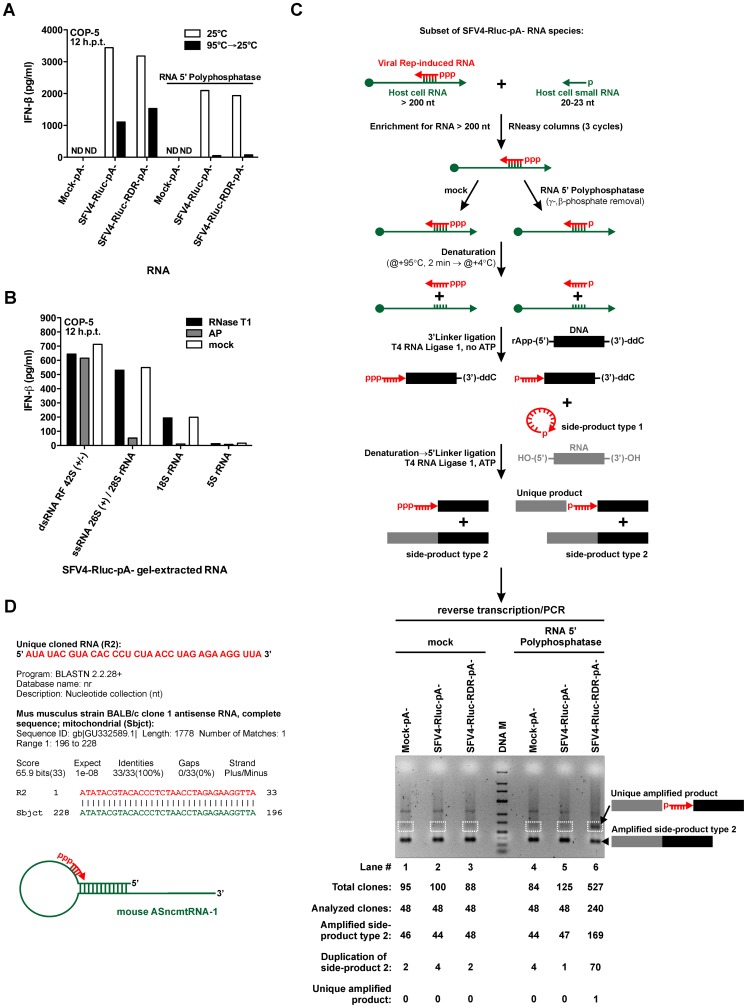
SFV replicase transcribes host cell RNA, triggering IFN-β induction during SFV infection. (A) Equal amounts (1 µg) of polyA− RNA fractions obtained by oligo(dT)-affinity chromatography fractionation from total RNA extracted from mock-, SFV4-Rluc-, and SFV4-Rluc-RDR-infected MEF cells were either treated (right) or not treated (left) with RNA 5′ polyphosphatase. Subsequently, RNAs were either denatured (black bars) or not denatured (white bars) and transfected into COP-5 cells. At 12 hr post transfection, the amount of IFN-β was determined in the cell culture medium by ELISA. ND, not detectable; h.p.t., hours post transfection. (B) SFV4-Rluc polyA− RNA fraction was resolved by performing electrophoresis on a native low melting 0.8% agarose gel and RNA species corresponding to viral dsRNA RF 42S (±), viral ssRNA 26S (+)/28S rRNA, 18S rRNA, and 5S rRNA were purified. Subsequently, obtained RNA species were mock-, RNase T1-, and alkaline phosphatase (AP)-treated and transfected into COP-5 cells, while the total RNA amount was kept constant with “stuffer” RNA (total RNA from naïve MEF cells). At 12 hr post transfection, the amount of IFN-β was determined in the cell culture medium by ELISA. h.p.t., hours post transfection. (C) Schematic of the strategy used to tag and amplify RNA fragments generated by SFV replicase (top). Obtained PCR products were resolved using electrophoresis on the 2% agarose gel (in tris-borate-edta buffer) (bottom). DNA M, DNA marker (top – 700, 500, 400, 300, 200, 150, 100, 75, 50, 25 – bottom, bps). (D) Unique cloned RNA identified using the strategy, shown in (C). Schematic representation of the stem-loop RNA is based on the sequence alignment (BLASTN 2.2.28+ [91]) of mouse ASncmtRNA-1 and human ASncmtRNA-2, for the latter such structure was experimentally confirmed [63]. (A) and (B) are representative of two independent experiments.

To further characterize PAMPs present in polyA− RNA fraction of SFV infected cells the importance of 5′-ppp structure and double-stranded nature of these RNAs were analyzed. We found that neither RNA denaturation nor γ- and β-phosphates removal from the RNA 5′-end alone did not diminish IFN induction substantially. Combination of RNA 5′-end phosphates removal with subsequent denaturation, however, resulted in almost complete loss of the IFN signal (Figure 8A). This result indicated that polyA− RNA fractions of infected cells contained the mixture of PAMPs. Second, to analyze the properties of these PAMPs in a more detail, the SFV4-Rluc polyA− RNA fraction was resolved by performing electrophoresis on a native low melting agarose gel and different RNA species were excised and extracted. Subsequently, obtained RNA species were treated with various enzymes and transfected into COP-5 cells (Figure 8B). RNase T1 treatment of the isolated RNA species did not have any effect on their capacity to trigger IFN-β, indicating that all analyzed fractions contained dsRNA. Alkaline phosphatase treatment, however, substantially reduced the IFN induction only for RNA species co-migrating with cellular 28S and 18S rRNA (Figure 8B). Importantly, similar structural properties (RNase T1 insensitivity, AP sensitivity) were observed for the IFN inducing RNA generated by Rep expression in COP-5 cells (Figure 3D).

Finally, to prove that SFV replicase indeed transcribes host cell RNAs producing 5′-ppp dsRNAs in the context of viral infection we attempted to identify an example of such RNA. Since IFN-inducing RNAs co-migrated with 28S and 18S rRNAs (Figure 8B) it was obvious that multiple cellular RNAs are recognized and used by viral replicase. Therefore an identification of any of such RNAs represents a considerable challenge. It was recently demonstrated that RIG-I activation depends critically on 5′-ppp structure only for a short dsRNA (∼40 bp), whereas increasing the length of the dsRNA compensates for the 5′-ppp removal [60]. Therefore the previous results indicate that we were searching for a 5′-ppp RNAs (Figure 8A–B), complementary to a polyA− host RNAs longer than 200 nucleotides and forming ∼40 bp dsRNA structure (Figures 7E and 8B). Consequently, we had to adopt the recently described method used for micro RNA (miRNA) cloning, based on sequential linker ligations to 3′- and 5′-ends of the miRNA. In this procedure, the ligation of the 3′-linker is a very efficient process, whereas the efficiency of 5′-linker ligation may differ 100-fold depending on the target miRNA [61]. Several additional difficulties, however, were associated with cloning of viral replicase-generated 5′-ppp RNAs. First, while 5′-p miRNA may have some secondary and tertiary structure, they are essentially single-stranded RNA molecules, whereas the 5′-ppp RNAs generated by the viral replicase form dsRNA structures. Second, every ligation step in miRNA cloning is verified by the electrophoretical mobility shift and ligated miRNA can be directly purified, whereas due to the underrepresentation (for example as compared to miRNA) and/or heterogeneity of 5′-ppp RNAs generated by SFV replicase such manipulations were not possible. Consequently, both ligation reactions were performed in a single tube (“one-pot synthesis”). Therefore the second and the most critical RNA denaturation step was not particularly efficient due to the presence of buffer and proteins. In addition, due to higher concentrations of reactants, intermolecular ligation of 3′- and 5′-linkers was clearly more efficient than ligation to desired RNA molecule. Furthemore, host cell small RNAs (including miRNAs), could also interfere with the cloning of the replicase-generated RNAs due to the presence of a 5′-monophosphate (α-phosphate).

Taking into account all these above listed considerations a procedure illustrated in Figure 8C was developed. Briefly, we first depleted polyA− RNA samples from small RNA species by three rounds of purification on size-exclusion silica columns. Subsequently these RNAs from mock-, SFV4-Rluc-, and SFV4-Rluc-RDR-infected cells were incubated in the reaction buffer either in the absence (negative control) or presence of the RNA 5′ polyphosphatase. The purified RNAs were subjected to sequential ligation with 3′- and 5′-linkers (Figure 8C). Because the 5′-ppp structure does not allow the ligation of the 5′-linker, no unique clones should be present in the control samples which were not treated with RNA 5′ polyphosphatase. After performing reverse transcription and PCR we detected a unique discrete amplicon of approximately 100 bp only for the RNA 5′ polyphosphatase-treated polyA− RNA fraction isolated from SFV4-Rluc-RDR-infected cells (Figure 8C, lane 6). In contrast, no specific amplification was observed for polyA− sample originating from SFV4-Rluc infected cell. Most likely this was caused by the inefficiency of the developed detection procedure.

The results indicated that either a population or a single RNA of ∼30–40 nucleotides were successfully amplified. Subsequently, we excised the gel fragment containing this ∼100 bp amplicon and the corresponding fragments from remaining samples for DNA purification and cloning into a plasmid vector. As expected considerably more clones were obtained for RNA 5′ polyphosphatase treated sample from SFV4-Rluc-RDR infected cells. To avoid possible biases approximately half of clones, obtained for each probe, were sequenced. The analysis of obtained sequences revealed that the majority of the clones contained an amplified side-product of type 2, likely present due to its trace amounts after gel purification (Figure 8C, bottom). Unexpectedly, we found an additional type of clones that contained two copies of side-product of type 2, linked by a bacterial sequence, suggesting that a recombination leading to duplication took place. Almost 30% of the clones containing this type of inserts were generated as a result of the specific amplicon cloning, whereas in other samples these inserts constituted only a small fraction (2–8%). Consequently, conventional cloning is not an efficient approach for the analysis of amplicons representing the products of viral replicase and other methods such as direct analysis of PCR products by deep sequencing should be used. Nevertheless, one unique clone was obtained for the RNA 5′ polyphosphatase treated sample from SFV4-Rluc-RDR infected cells, providing an example from the putative population of ∼30–40 nucleotide RNAs (Figure 8D). This clone (named R2) contained a 33-nt copy of RNA, which was perfectly complementary to mouse antisense non-coding mitochondrial RNA 1 (ASncmtRNA-1, GenBank: GU332589.1), an equivalent of human ASncmtRNA-2 transcript (GenBank: EU863790.1) [62], [63], [64], [65]. The function of the human ASncmtRNAs is unknown, however it was demonstrated that these transcripts, co-migrating with the 18S rRNA and having a stem-loop structure, are down-regulated in tumor cell lines and tumor cells present in 17 different tumor types [63]. Furthermore, the possibility that R2 clone corresponded to a fragment of 16S rRNA encoded by mitochondrial precursor RNA (GenBank: V00665.1) was excluded due to the following reasons. First, the presence of ∼30 nt RNA fragment corresponding to 16S rRNA 3′-terminus would indicate that the integrity of RNA had been compromised. This was, however, highly unlikely since no ribonuclease activity was detected in either of the components of the reaction mixtures used for RNA enrichment and tagging (Figure 8C). Second, if R2 would have resulted from normal cellular RNA rather than from RNA containing 5′-ppp generated by SFV replicase, amplicons of corresponding size and similar clones should have been detected also for other samples, which was not the case. Third, R2 clone has an extra adenosine residue at its 3′-end (…GUUA) that is not present at the 3′ terminus (…GUU) of the major processed form of 16S rRNA [66], [67], indicating the absence of a perfect match between these sequences. Fourth, minor processed forms of the mouse 16S rRNA have GUUAU, GUUAUU, GUUAUUAGG, or GUUAUUAGGG sequences at their 3′-termini [66], however, these sequences were not present in R2 clone either. Finally, it was demonstrated that a small fraction of 16S rRNA contained polyA tails [66], [68] and was recoverable either by oligo(dT)-affinity chromatography [68] or by RT PCR [69], indicating that the presence of the polyA tail consisting of a single adenosine in the 16S rRNA is highly unlikely.

Moreover, the R2 clone corresponded to all PAMP criteria listed above – in addition to having expected length it was complementary to host polyA− RNA longer than 200 nt. Accordingly, as there is no cellular RdRp activity capable for synthesis of such RNA, these results strongly suggest that such RNA was synthesized by viral replicase. Moreover, the fact that we were unable to obtain this amplicon without the use of RNA 5′ polyphosphatase treatment indicated that this RNA had also 5′-ppp structure. Thus, an RNA molecule which represents an example of SFV replicase generated PAMP was experimentally identified. Based on our results (Figure 8B), it is also logical to assume that it does not represent the only PAMP of this kind generated by SFV replicase and its relative contribution to observed IFN induction is currently unknown. This as well as identification of full spectrum of sequences of SFV replicase generated PAMP RNAs represents topic for additional studies.

## Discussion

To subvert the vertebrate innate immune system, RNA viruses have evolved many different strategies. One of the most efficient of these strategies consists of blocking access of host sensors to viral dsRNA or 5′-ppp RNA. In the case of negative-strand RNA viruses and viruses with a dsRNA genome, the viral particles contain a replicase complex with genomic RNA. By retaining both their genomes and replicase in partially uncoated viral particles, negative-strand RNA viruses ensure that viral 5′-ppp RNA is not accessible to RLRs and that no exogenous (host cell) RNA can be transcribed. However, in the case of positive-strand RNA viruses, the viral particles deliver only viral genomic RNA into the cell, which serves as the mRNA for the synthesis of the viral replicase [70]. Therefore, it remains possible that host cell RNA could be transcribed by the viral replicase of positive-strand RNA viruses. At a later time in infection cycle, positive-strand RNA viruses remodel host cellular membranes to accommodate their replication complexes and thereby shield their own 5′-ppp dsRNA [71], [72].

In this study, we report a novel mechanism by which positive-strand RNA viral infection is sensed by the innate immune system. To demonstrate the importance of this mechanism for virus infection, we also describe a mechanism by which MEFs restrict the replication of a non-pathogenic SFV-Rluc-RDR. While others have shown that the mutant virus triggered increased IFN-β induction, we also demonstrated that this led to the destruction of positive-strand viral RNAs and the shutdown of viral replication in primary MEFs (Figure 5). Thus, IFN-induction is undoubtedly important for limiting SFV infection. Here, we delineate the mechanism that mediates this important antiviral response.

**Figure 9:**
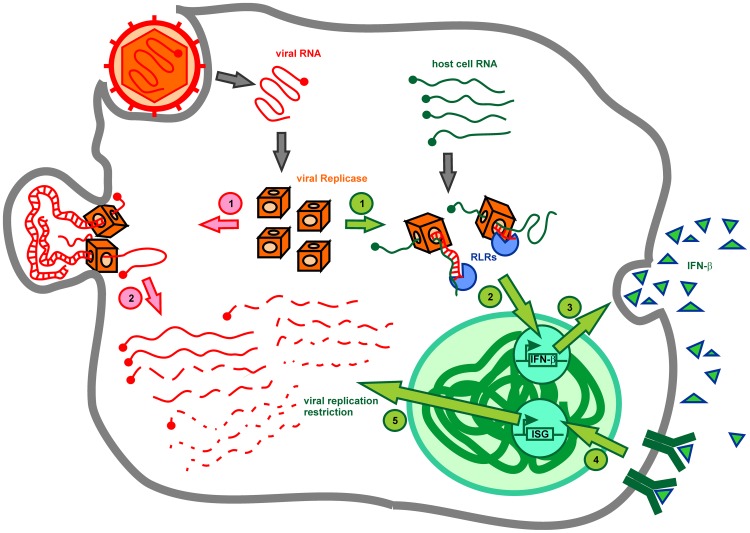
Model of mutant SFV replication restriction in fibroblasts. Transcription of the host cell RNA by the SFV replicase leads to generation of 5′-ppp dsRNA, which is detected by RLRs (RIG-I and MDA-5). Subsequently, type I IFN is induced and secreted. Released type I IFN activates transcription of interferon-stimulated genes (ISG), which trigger viral replication restriction by degrading SFV genomes.

To understand the mechanism by which alphaviruses trigger potent IFN-β production, we dissected the replication of SFV by decoupling the expression of the replicases of the wild-type and mutant viruses from their viral replication-competent RNA templates. First, it was shown that the replication of the short virus-like RNA template by these replicases triggered the production of IFN-β by COP-5 cells (Figure 1C). However, it was subsequently found that these replicases were fully capable of IFN-β induction even when expressed in the absence of any virus or virus-like RNA template (Figure 1D). In both cases, the mutant replicase induced increased IFN-β production, as compared to the wild-type replicase, and we observed that the viral replicase RdRp activity was responsible for IFN-β induction (Figures 1D–E). Moreover, we found that only the viral replicase with intact RdRp activity generated IFN-inducing RNA, which triggered the IFN-β production (Figure 3A). Our results strongly suggest that during SFV infection, two parallel processes are driven by the viral replicase; in the first process, the viral replicase drives the replication and transcription of the replication competent viral genome, whereas in the second process, the viral replicase drives the transcription of non-viral host cell RNA templates (Figure 9).

The IFN-inducing activity of the viral replicase-generated RNAs was resistant to RNase A and RNase T1, although this activity was sensitive to RNase III and alkaline phosphatase, which indicated that this ligand is a dsRNA containing at least one terminal phosphate (Figure 3D). Our interference experiments indicated that RIG-I was the primary sensor detecting IFN-inducing RNA produced by the SFV replicase (Figure 2B). This result also strongly suggests that this RNA contains a 5′-ppp. The immunofluorescence results indicated that IFN-inducing RNA generated by the SFV replicase contained duplex RNA regions exceeding 40 bp in length (Figures 4B and 4C). Thus, SFV replicase transforms host cell RNA into PAMPs that are recognized and counteracted as non-self entities. This strategy of innate immune recognition of a viral replicase is different from pattern recognition as the replicase generates novel PAMP structures not uniquely associated with a pathogen. Detection of viral replicase RdRp activity by vertebrate host cells may represent an ancient mechanism for the recognition of non-self enzymatic function.

The majority (∼90%) of the IFN-β-inducing RNA generated by the SFV replicase was non-polyadenylated (Figure 3B). In this regard, it is interesting to note that non-polyadenylated transcripts comprise almost half of the human and mouse transcriptomes, although the biological function of these transcripts remains unclear [73]. Therefore, it will be of significant interest to identify which non-polyadenylated transcripts encoded by the host genome are involved in viral replicase detection. Our results also exclude the possibility that SFV replicase-triggered IFN-β induction is mediated by the antiviral endoribonuclease RNase L, which has been reported to cleave host cell RNA to generate small RNAs with lengths less than 200 nt upon viral infection [45]. In fact, we found that host cell RNAs longer than 200 nt are modified by SFV replicase to induce IFN-β (Figure 3A). Subsequently, identical characteristics were observed for the PAMPs present in the non-polyadenylated RNA fractions isolated from SFV-infected MEF cells (Figure 7D,E).

Consistent with previously published results obtained using bone marrow-derived dendritic cells [32], we demonstrated that MEFs detect SFV using the MDA-5 and RIG-I sensors (Figure 2C). We observed that detection of SFV4-Rluc-RDR heavily relied on both MDA-5 and RIG-I, whereas for detection of the viral replicase, RIG-I was the primary sensor (Figure 2B). This discrepancy is likely due to the fact that long viral dsRNA replication intermediates are generated during SFV infection. It was previously demonstrated that MDA-5 and RIG-I detect long and short dsRNAs, respectively [5], and these findings further support the idea that the viral replicase drives both viral RNA replication and transcription using host cell RNA templates. In both cases the predominant type of RNA ligand produced by SFV replicase dictates the primary type of sensor responsible for ligand detection and IFN induction.

Due to codon-optimization, the mRNA encoded by pRep and pRep-RDR lacked any cis-sequences that are known to participate in SFV RNA replication; hence, the PAMPs generated in the pRep-RDR transfected cells were non-viral. More importantly, our study reveals that host cell RNAs were used as alternative templates by the viral replicase during virus infection, demonstrating for the first time that during positive-strand RNA virus infection, both viral RNAs and cellular RNAs that are modified by the viral replicase contribute to IFN-β induction. The latter conclusion is based on the following data. Fractionation of total RNA from infected primary MEF cells by oligo(dT)-affinity chromatography led to a 15-fold enrichment of the major viral RNA species in the polyadenylated RNA fraction compared with the non-polyadenylated RNA fraction (Figure 7A,B). Consequently, if the viral RNA is the only PAMP present in infected cells, then the polyadenylated RNA fraction should induce a 15-fold higher level of IFN-β compared with an equal amount of the corresponding non-polyadenylated RNA fraction. Remarkably, this was not the case, as polyadenylated RNA fractions from wild type and mutant virus infected cells induced only approximately 2-fold and 6-fold higher levels of IFN-β respectively (Figure 7C). These findings strongly suggest that non-polyadenylated RNA fractions isolated from infected MEF cells contain potent IFN-β inducers, most of which are not viral RNAs and were derived from the host cell RNA. Strikingly, this finding was especially compelling for the non-polyadenylated RNA species extracted from the MEF cells infected with the wild type virus. These results cannot be explained based on the current paradigm that type I IFN is triggered exclusively by viral dsRNA or viral RNA containing a 5′-ppp that are produced during the course of viral genome replication or transcription by viral replicases [5], [6], [12], [74], [75], [76].

Our results indicate that wild type and mutant SFV generate roughly the same amount of PAMPs. However, the amount of IFN-β that is induced by these viruses in MEF cells differs drastically (Figure 6) and, in the case of mutant virus, leads to the inhibition of viral replication (Figure 5). Obvious explanation to this conundrum is the presence of a single mutation in the nsP2 subunit of the viral replicase of SFV4-Rluc-RDR, which does not allow nsP2 to enter the nucleus of infected cell. It has been reported that the nuclear localization of nsP2 (for both SFV and SIN) is absolutely required for the suppression of the host cell antiviral response [20], [21], [27], [28]. Consequently, it is thought that nsP2 must suppress the innate immune system of the host cell to inhibit the sensing of the viral RNA PAMP(s) and/or response to the PAMPs. However, there are several arguments that challenge this hypothesis. First, cytosol-accessible SFV genomic (42S [+] strand) and subgenomic (26 [+] strand) viral RNAs, which direct the synthesis of viral replicase and viral structural proteins, are not PAMPs because their 5′-ppp is shielded by a cap-structure [55]. Second, the PAMP represented by the dsRNA replicative form (42S [±]) resides in a membrane-bound replication complex [48], [77]. Third, the 42S (−) strand, which does not have cap-structure [78] and, thus, may represent a PAMP, most likely does not exist as a free molecule (Figure 7B); instead, this strand is confined to the replication complex as part of the dsRNA. Indeed, our data clearly indicate that this RNA does not exist as a free molecule, as it was fully protected in the SFV4-Rluc-RDR infected MEF cells and, unlike free positive-strand RNA, was not degraded during the restriction of viral replication (Figure 5J). Thus, it appears that RLR sensors may have extremely limited access to viral PAMPs in infected cells. The only species that are presumably accessible to RLRs are DI-RNAs, which have a negative polarity and are double-stranded. However, although DI-RNA is presumably localized to the spherules, this has not been experimentally verified. DI-RNA may also contribute to IFN induction, but this contribution is likely to be minor, as their amount was found to be negligible compared with the full-length viral RNA species (Figure 7B). Moreover, the amounts of SFV DI-RNA produced during both wild type and mutant virus infection are very similar (Figure S4) and cannot explain the dramatic difference in IFN-β induction (Figures 5B and 6B). Still, a mutation in nsP2 results in the rapid detection of SFV4-Rluc-RDR infection (Figure 6B) and the restriction of viral replication by the host cell (Figure 5). Consequently, there are PAMPs that are easily accessible to RLR sensors in infected MEF cells. Our study strongly suggests that these easily accessible PAMPs are generated by viral replicase unspecific activity on host cell RNAs.

It is known that enzymatically active nonstructural viral proteins that participate in the formation of the viral replicase complex are produced in much larger amounts than are needed for virus RNA replication. This has been demonstrated for alphaviruses [36], [47] and HCV [79]. It has been proposed that these viral proteins play roles in processes other than viral RNA replication [36]. For example, the NS3/4A protease of HCV cleaves mitochondrial antiviral protein, precluding HCV detection by the host cell [80]. Proteases of picornaviruses cleave multiple host factors, including eIF4G, precluding the translation of host cell capped mRNAs [81]. Though the list of the enzymatic activities of viral proteins that target host cell components is long and rapidly growing, it is clearly biased towards the activities that are beneficial for virus infection. At the same time, it is hard to believe that this process is one-sided. The host cells have also evolved to take advantage of some of these enzymatic functions specific to viruses. In this regard, the use of these activities to detect the presence of a pathogen is a clear possibility that has not been considered. There are many benefits to recognizing RdRp activity of viral replicases rather than viral RNA PAMPs. First, especially in the case of in vivo infections, not all of the cells are permissive for viral RNA replication. However, entry of viral RNA into such cells will trigger the production of viral replicase, the RdRp activity of which then can be detected by the host cell. Second, all RNA viruses inevitably produce divergent progeny, including many viruses that are replication defective, which are also packed into virions. As long as the defective RNAs are capable of expressing a functional replicase, the synthesis of nonviral PAMP RNAs will occur.

In this study, we have developed a procedure that allowed us to selectively amplify the RNAs produced by the SFV replicase in the context of SFV4-Rluc-RDR infection (Figure 8C). However, because of the inefficient conventional cloning approach only a single non-polyadenylated RNA used by SFV replicase was identified (Figure 8D). It has recently been demonstrated by deep sequencing analysis that heterogeneous host cell RNAs (22 mRNAs, 3 non-coding RNAs, and 2 pseudogenes) are modified by the HCV RdRp when expressed alone in the mouse liver [41]. Our data strongly suggests that non-polyadenylated RNAs co-migrating with 28S and 18S rRNAs are the primary targets for the SFV replicase (Figures 3B, 7C, 7E, 8A, and 8B). We demonstrate that PAMPs produced from host cell RNAs are an important byproduct of alphavirus replicase (nsP1/nsP2/nsP3/nsP4) RdRp activity during infection and that the nsP2 protease subunit is used to counteract the consequences of this activity. For HCV, the activity of NS5B RdRp is counteracted by NS3/4A protease [13], [82], [83]. To date, the replicases of HCV, norovirus, and TMEV have been shown to be capable of inducing IFN without the requirement of viral RNA replication [13], [14], [15], [16]. Direct comparison of the IFN induction by SFV replicase and HCV RdRp showed that both replicases are very efficient PAMP inducers (Figure 1F). Therefore, together with our study, these findings suggest that the activation of the innate immune response by the replicases of positive-strand RNA viruses may be a general property used by the host cells to counteract viral invasion. If so, the question of why viruses have not evolved a way for their replicase to be more specific for their own RNAs remains. The likely reason is that the increased specificity of the replicase is not beneficial for the survival and/or evolution of the virus. Alphavirus replicases possess an outstanding ability to recognize defective cis-elements, repair such defects and rescue the replication in their genomes [57]. Thus, even if it were possible to obtain a polymerase with higher template specificity, the cost would be too high. This theory parallels reports studying the error prone nature of the same viral RNA replicases. As it was elegantly demonstrated, the fidelity of the picornavirus RNA polymerase can be easily increased [84], but this increased fidelity results in reduced virus fitness and virulence [85]. Therefore, as discussed above, viruses have instead armed themselves with safeguards that are effective against innate immune recognition irrespective of the origin of the PAMPs.

## Materials and Methods

### Plasmids, Antibodies, and Reagents

pRep was generated by inserting codon-optimized coding sequence of SFV replicase (EMBL-Bank: HC198689) into either KS plasmid (Stratagene) or GTU eukaryotic plasmid expression vector [86]. To generate pRep-RDR, we introduced mutations C4129G, G4130A, and G4131C (nucleotide numbering as in HC198689) leading to RRR→RDR mutation in nsP2 nuclear localization sequence of SFV replicase. To obtain pRep-RDR/GAA plasmid DNA, we introduced additional mutations A7424C and A7427C, inactivating the nsP4 RdRp catalytic centre (GDD→GAA). pRep, pRep-RDR, and pRep-RDR-GAA contained intron sequence from rabbit beta-globin gene (GenBank: V00882.1), which was inserted into SFV replicase coding sequence. The expression of SFV replicase polyprotein was under control of either hEF1α/HTLV composite promoter (increased expression) or Rous sarcoma virus long terminal repeat (RSV LTR). For the generation of pNS5B and pNS5B-GND, the coding sequence of NS5B Con1 genotype 1b (GenBank: AJ238799) was used. To keep total DNA amount constant during dose-dependent IFN-induction experiments, transfections were performed with “stuffer” DNA plasmid vector encoding d1EGFP.

Plasmid pSFVmin was constructed from two fragments. First fragment of SFV cDNA corresponding to the nucleotides 1–274 of SFV genome was placed under control of CMV promoter and cloned into KS plasmid (Stratagene). Second fragment consisting form polylinker (BstB1-PmeI-BglII-SpeI-NotI), complete cDNA copy of 3′ UTR of SFV4 with polyA sequence of 60 residues, hepatitis delta virus negative strand ribozyme and SV40 early transcription terminator was cloned immediately downstream of the first fragment. To obtain pSFVminRluc the coding sequence of Rluc was amplified by PCR and inserted to pSFVmin using SpeI-NotI restriction sites. In the resulting construct pSFVminRluc Rluc reporter is expressed in form of fusion protein containing 78 foreign aa residues at its N-terminus, first 63 of them representing the N-terminal region of nsP1 of SFV.

The antibodies for mouse RIG-I (R37) and LGP2 were purchased from Immuno-Biological Laboratories Co., Ltd. The antibody for mouse MDA-5 (AL180) was purchased from Alexis Biochemicals. The antibody for dsRNA (J2) was purchased from Scicons. Antibody for β-actin (C4) was purchased from Santa Cruz Biotechnology, Inc. The antibody for LAMP2 (H4B4) was purchased from Abcam. SFV nsP2 and nsP4 antibodies were kindly provided by Dr. Tero Ahola, antibodies against nsP1 and nsP3 of SFV were made in-house. Secondary antibodies used in immunofluorescence were purchased from Life Technologies.

Poly(I:C) and chloroquine were purchased from Sigma-Aldrich. RNase A, RNase T1, and RNase III were purchased from Ambion. Alkaline phosphatase and DNase I were purchased from Roche Applied Science and Promega respectively. T4 RNA Ligase I and β-Agarase I were purchased from New England Biolabs. RNA 5′ polyphosphatase was purchased from Epicentre (Illumina).

### Cell Culture and Transfection

COP-5 and RD cells were maintained in L-glutamine-containing IMDM medium supplemented with 10% fetal bovine serum (FBS), and antibiotics. Primary MEF cells were purchased from Millipore (EmbryoMax Primary Mouse Embryo Fibroblasts, Not Mytomycin C Treated, Strain CF1, passage 3; Catalogue Number: PMEF-CFL) and cultured in L-glutamine-, sodium pyruvate-, and high glucose-containing DMEM medium supplemented with 15% FBS, 0.1 mM β-mercaptoethanol, and antibiotics up to passage 6. Transfection of DNA (0.2 µg/ml) and RNA (0.2–0.4 µg/ml) into COP-5 cells (0.25–1.0×10^6^ per 60-mm dish) was carried out using Lipofectamine 2000 (Invitrogen). RD cells (0.2×10^6^ per 60-mm dish) were transfected with DNA (0.2 µg/ml) by electroporation in GenePulser Xcell (Bio-Rad) instrument (settings: Exponential Wave, 190 V; 975 microfarads [µF]) in 400 µl OptiMEM medium (Invitrogen). RNA (2 µg/ml) and DNA (10 µg/ml) into MEF cells (0.25×10^6^ per 60-mm dish) were transfected by electroporation (Exponential Wave, 240 V; 975 µF) in 400 µl OptiMEM medium. All electroporations were performed in 4-mm cuvettes (Thermo Fisher Scientific).

### Viral Infection

SFV4-Rluc and SFV4-Rluc-RDR were constructed, rescued and propagated as previously described [44]. The viral stocks were titrated using plaque titration on baby hamster kidney (BHK)-21 cells. For the infection of MEFs, which are considerably less susceptible to SFV4 infection than BHK-21 cells, the relative titers and MEF-specific MOIs were re-calculated based on the infectivity of the recombinant viruses, as measured by immunostaining of infected cells. The viral infection of primary MEFs was performed in OptiMEM supplemented with 0.1% FBS for 1 hr. The virus was subsequently aspirated, and fresh medium was added.

### Infectious Center Assay

An infectious center assay was performed essentially as previously described with minor modifications [87]. Briefly, 1 µg of RNA fractions extracted from infected MEF cells were electroporated (two pulses 850 V, 25 µF, in 800 µl) into BHK-21 (baby hamster kidney) cells. Tenfold dilutions of electroporated cells were seeded into six-well plates containing monolayers of naïve BHK-21 cells. After a 2 hr incubation at 37°C, the cell culture medium was aspirated, and the wells were overlaid with a medium containing carboxymethyl cellulose. After 2–3 days, the plaques were visualized by crystal violet staining and counted.

### ELISA

The amount of IFN-β secreted into the cell culture medium was measured using a commercial Verikine Mouse Interferon-Beta ELISA kit (PBL InterferonSource), according to the manufacturer's instructions.

### Renilla Luciferase Reporter Assay

Cells were harvested (scraped with a rubber policeman) at different time points in a phosphate-buffered saline (PBS) on ice. Subsequently, cells were lysed with Renilla Luciferase Assay Lysis Buffer according to the manufacturer's instructions (Promega). Lysate was mixed with Renilla Luciferase Assay Substrate diluted 100-fold in Renilla Luciferase Assay Buffer, and luminescence was measured on the GloMAX 20/20 Luminometer (Promega).

### Design of siRNAs

We used the algorithm developed in-house to design 21-nt siRNA oligos, which had 19-bp perfect match duplex and 2-nt 3′-overhangs. The sequences of siRNA oligonucleotides used in the study are as follows (siRNA duplex name, guide strand [5′→3′], passenger strand [5′→3′]): dhx58_mus_2304, UUCUUAGAACAUCAUGGCAUA, UGCCAUGAUGUUCUAAGAACU; ifih1_mus_3004, AUUGACAUGAUGCAUCUUCUC, GAAGAUGCAUCAUGUCAAUAU; ddx58_mus_2678, AUAUCUUCCACGACGAAACUU, GUUUCGUCGUGGAAGAUAUUG. siRNA duplexes were synthesized and annealed by Proligo (Sigma-Genosys), whereas negative control non-targeting siRNA #4611 and #4635 was purchased from Ambion.

### RNA Interference

siRNA oligonucleotides at a final concentration of 20 nM were reverse-transfected into COP-5 cells (0.25–1.0×10^6^ cells per 60-mm dish) using 5 µl Lipofectamine RNAiMAX (Invitrogen). For primary MEFs (0.25×10^6^ cells per 60-mm dish), siRNA duplexes at a final concentration of 100 nM were transfected by electroporation in 4-mm cuvettes (Thermo Fisher Scientific) using a GenePulser Xcell (Bio-Rad) instrument (settings: Square Wave, 1000 V; 2 pulses, 0.5 ms) in 100 µl OptiMEM medium. Unless otherwise indicated, on the third day of culture, cells were transfected with 0.2 µg/ml of plasmid DNA or RNA using 5 µl Lipofectamine 2000 (Invitrogen) or infected by SFV4-Rluc-RDR. The amount of IFN-β in the cell culture medium was measured, and cells were harvested for the immunoblotting analysis on either the fourth or fifth day of culture.

### Immunoblotting

Proteins in cell extracts were resolved on 10% polyacrylamide/SDS gels in Mini PROTEAN Tetra Cell systems (Bio-Rad). Subsequently, proteins were transferred to Immobilon-P (Millipore) polyvinylidene fluoride microporous 0.45 µm membranes using Trans-Blot Semi-Dry Transfer Cell apparatus (Bio-Rad). Blots were incubated with various primary antibodies. Secondary goat anti-rabbit and anti-mouse antibodies conjugated with horseradish peroxidase were from LabAs Ltd. Immunoreactive bands were detected by enhanced chemiluminescence (ECL) (GE Healthcare) and subsequent exposure to X-ray film (SuperRX, Fuji).

### Nucleic Acid Extraction

Total RNAs, large RNAs (>200 nt), and small RNAs (<200 nt) were extracted from cells and purified using the *mir*Vana miRNA Isolation kit (Ambion) or TRIzol reagent (Invitrogen), according to the manufacturer's instructions. Oligo(dT)-Cellulose Type 7 (GE Healthcare) was used for affinity-chromatography fractionation of total RNA into polyadenylated (polyA+) and non-polyadenylated (polyA−) RNA species. For RNA extraction from native low melting agarose gel, β-Agarase I enzyme was used accordingly to manufacturer's protocol (New England Biolabs). When required, PD-10 desalting columns (GE Healthcare) containing Sephadex G25 were used for buffer exchange. The resulting OD_260_/OD_280_ and OD_260_/OD_230_ ratios for all RNA preparations exceeded 2.1, as determined by measurements obtained using a ND-1000 spectrophotometer (NanoDrop Technologies, Inc.). The integrity of the RNA was confirmed by denaturing formaldehyde agarose gel electrophoresis. Alternatively, RNA was resolved on non-denaturing agarose gel electrophoresis, stained with ethidium bromide, its image was recorded and analyzed by NIH ImageJ 1.46 software (http://rsb.info.nih.gov/ij/download.html).

### Enzyme Treatments of Nucleic Acids

Two micrograms of nucleic acid were treated with DNase I (0.1 U/µl) and alkaline phosphatase (0.1 U/µl) at 37°C and 50°C, respectively, for 1 hr in a volume of 20 µl. For RNase digestion experiments, 2 µg of RNA was digested with RNase A, RNase III, or RNase T1 at the specified concentrations at 37°C for 1 hr in a volume of 20 µl. The undiluted (1×) RNase concentrations used in the reactions were 1 µg/ml (RNase A), 1 U/µl (RNase III), and 1 U/µl (RNase T1). Enzyme-treated RNAs were precipitated with ethanol in the presence of sodium acetate and glycogen prior to transfection.

### RNA Extraction from Sub-cellular Fractions of COP-5 Cells

Sub-cellular fractionation was performed as previously described [88]. In brief, pRep-RDR or pRep-RDR/GAA transfected COP-5 cells (4–6×10^7^) were harvested, washed, and resuspended in 800 µl of HB buffer (8.6% sucrose, 3 mM Imidazole, pH 7.4) supplemented with protease inhibitors (Roche). Subsequently, cells were homogenized by passing homogenate through 22G1 ¼ needle mounted onto 1-ml syringe until the ratio of unbroken cells to free nuclei was 10% to 90%, as examined under microscope. Unbroken cells and nuclei were pelleted by centrifugation and post-nuclear supernatant (PNS) collected. Concentration of sucrose in the PNS was adjusted to 40.6% using 62% sucrose solution and refractometer. PNS was loaded in the bottom of an SW41 centrifuge tube and overlaid with 4.5 ml of 35% sucrose, 3 ml of 25% sucrose, and 3 ml of 8.6% sucrose cushions. All sucrose cushions also contained 3 mM Imidazole pH 7.4 and 1 mM EDTA. Tubes were centrifuged for 1.5 hr at 35000 rpm in a Beckman Optima L-90 K ultracentrifuge at 4°C. After centrifugation the pellet, containing cytosolic ribonucleoprotein complexes and whitish bands of membrane particles at every interphase between sucrose cushions were collected. Subsequently, RNA was extracted from each fraction with TRIzol Reagent (Invitrogen). Before isopropanol precipitation 40 µg of RNA-grade glycogen was added to each sample for maximal recovery of RNA.

### Immunofluorescence

COP-5 or RD cells transfected with pRep, pRep-RDR, or pRep-RDR/GAA were washed twice with phosphate-buffered saline (PBS), fixed with 4% paraformaldehyde in PBS for 10 min at 22°C, and permeabilized with 0.5% Triton X-100 in PBS for 5 min at 22°C. Blocking and antibody binding was performed in two different ways. First, samples were treated with block buffer (10% goat serum and 1% BSA in PBS) for 1 hr at 22°C, and then incubated for 1 hr at 22°C with antibodies against LAMP2 (H4B4, Abcam) and nsP1 diluted in antibody-binding buffer (3% BSA and 0.05% Tween 20 in PBS). Second, samples were incubated in block buffer (10% goat serum, 1% BSA, and 0.2% Triton X-100 in PBS), and consequently with antibodies against dsRNA (J2) and nsP1 in 3% BSA, 0.2% Triton X-100, and 10 mM MgCl_2_ antibody-binding buffer. Antibody binding was detected using appropriate antibodies conjugated with Alexa fluor 488 and 568 (Invitrogen). Specifically, for H4B4 and J2 binding detection, secondary antibodies purchased from Life Technologies and reacting with the Fc portion of the heavy chain of mouse IgG1 (A-21121) and IgG2a (A-21134) were used respectively. SlowFade Gold antifade reagent with DAPI (Invitrogen) was used for counterstaining of cells nuclei. Samples were imaged on a Nikon ECLIPSE TE2000-U inverted microscope and recorded with Nikon DXM1200C Digital Camera. Images were collected using 60× immersion objective and processed with Nikon Capture NX2 and ACT-1C software.

### Oligo(dT)-Affinity Chromatography

Oligo(dT)-Cellulose type 7 powder (GE Healthcare) was suspended in sterile water and resulting gel was used for the preparation of gravity-flow chromatography columns. Subsequently, oligo(dT) columns were washed with 10 volumes of water and 5 volumes of 100 mM sodium hydroxide (pH∼10). The pH of the oligo(dT)-columns was brought to 7.5 by equilibrating it with 10 volumes of TEN0 buffer (10 mM TrisHCl, 1 mM EDTA, pH 7.5) and subsequently with 10 volumes of TEN500 (10 mM TrisHCl, 500 mM sodium chloride, 1 mM EDTA, pH 7.5). Total RNA samples were heat-denatured (70°C, 10 min) in water, chilled on ice and loaded onto columns in TEN500 buffer. Then, unbound nonpolyadenylated RNAs were collected. Oligo(dT) columns with bound polyadenylated RNAs were extensively washed with 30 volumes of TEN500 buffer. Finally, bound RNA was eluted with 10 volumes of TEN0 and RNA-containing fractions were pooled.

### Northern Blotting and In Vitro Transcription

RNA samples were denatured in loading buffer (1×MOPS, 50% formamide, and 6% formaldehyde) for 5 min at 100°C, chilled on ice and separated on 1% agarose 6% formaldehyde-containing denaturing gel using 1×MOPS buffer system (5 V/cm, 5 hr, 4°C). Consequently, samples were transferred to nylon membranes (Hybond N+, GE Healthcare) in 10X SSC using capillary blotting technique for 16 hr. After UV cross-linking at 0.12 J/cm^2^ the membrane was blocked in hybridization solution (water-reconstituted DIG Easy Hyb Granules [Roche]) at 65°C for 30 min in the hybridization vessels. The RNA probes were generated with phage T7- and T3-RNA polymerases run-off in vitro transcription using linearized DNA as templates and labeled with digoxigenin-UTP-containing RNA labeling mix (Roche). Consequently, RNA probes were treated with DNase I to remove template DNA, purified on Illustra S-300HR Columns (GE Healthcare), denatured and added to the hybridization vessels at 100 ng/ml for hybridization at 65°C for 18 hr. The membranes were washed twice in 2X SSC, 0.1% SDS for 10 min at 25°C and then twice in 0.1X SSC, 0.1% SDS for 15 min at 65°C. Consequently, membranes were washed, blocked, and incubated with Fab fragments against digoxigenin, conjugated to alkaline phosphatase for detection of hybridized signals using CDP-*Star* (DIG Luminescent Detection Kit, Roche Applied Science) according to manufacturer's instructions. Finally, membranes were exposed to SuperRX X-ray films (Fuji) or for longer exposure times in ImageQuant RT ECL system (GE Healthcare).

Model dsRNA for RNase digestion experiment was generated by annealing of two single-stranded RNAs generated by T7 and SP6 RNA polymerases via run-off transcription from the plasmid DNA containing hepatitis C virus subgenomic replicon sequence fragment (∼4000 bp) flanked by corresponding promoters. RNAs were dissolved in RNA annealing buffer (10 mM TrisHCl, 20 mM NaCl, pH 7.5), denatured for 1 min at 98°C, then incubated at 75°C for 10 min, and finally cooled to room temperature during 1 hr.

### Alkaline Hydrolysis of DIG-labeled RNA

DIG-labeled full length SFV4-Rluc RNA was incubated in 1× alkaline hydrolysis buffer (50 mM NaHCO_3_/Na_2_CO_3_, 1 mM EDTA, pH 9.2) at 95°C for 6 min and rapidly cooled to 4°C. Subsequently, fragmented RNA was purified using RNeasy Mini Kit (QIAGEN).

### Tagging of the SFV Replicase Generated RNA

Small RNA species (∼20–23 nt) were depleted from polyA− RNA samples by three rounds of purification using RNeasy Mini Kit (QIAGEN). Subsequently RNAs were incubated in the reaction buffer either in the absence (negative control) or presence of the RNA 5′ polyphosphatase (Epicentre, Illumina). After purification using RNeasy Mini Kit (QIAGEN), the RNAs were subjected to sequential ligation with pre-adenylated and blocked at its 3′-terminus full-DNA 3′Linker (IDT Linker-1, 5′-rApp-CTG TAG GCA CCA TCA AT-ddC-3′, Integrated DNA Technologies) and full-RNA 5′Linker (5′-GCC ACC UCG AGU CAC ACC GUA AGU UUC-3′ [89]) essentially as described previously with minor modifications [61]. First, we used T4 RNA Ligase 1 (New England Biolabs) for both steps. Second, both ligation reactions were performed in a single tube (“one-pot synthesis”). Third, after second denaturation step additional ligase was added. Ligation mixtures were used directly for reverse transcription and PCR.

### Reverse Transcription (RT) and PCR

RNA (100 ng) was reverse-transcribed using the SuperScript III First-strand Synthesis System for RT-PCR (Life Technologies) in a final volume of 20 µl according to the manufacturer's instructions with minor procedure modifications. Briefly, the RNA, the primer, and dNTPs were incubated for 2 minutes at 95°C and cooled quickly on ice. The remaining components (reverse transcriptase buffer, reverse transcriptase, MgCl_2_, dithiothreitol, RNase inhibitor, and water) were mixed and added on ice. The reactions were initiated by shifting the temperature to 50°C for 2 hr and stopped by heating at 85°C for 5 minutes. Subsequently, RNAs were removed by RNase H treatment. Two microliters of the RT reaction mixture was used for subsequent PCR analysis, which was performed either with the Phusion or with the Dynazyme II polymerases (Thermo Scientific) according to the manufacturer's instructions. For strand-specific RT, we used the HPLC-purified primers (Microsynth) 5′SFV (5′-ATG GCG GAT GTG TGA CAT ACA CGA C-3′) and 3′SFV (5′-GGA AAT ATT AAA AAC CAA TTG CAA AAT AAA ATA-3′) as previously described to efficiently amplify SFV DI-RNA [59] for negative and positive strand detection, respectively. Both primers were used for PCR amplification of the RT products. For the amplification of cDNA, corresponding to tagged SFV replicase generated products, we used HPLC-purified primers Y-adaptor-a (5′-GCC ACC TCG AGT CAC ACC GTA-3′) [89] and AF-JIG-37 (5′-CAA GCA GAA GAC GGC ATA CGA ATT GAT GGT GCC TAC AG-3′) [90], the latter primer was also used for RT.

## Supporting Information

Figure S1 **Expression of SFV replicase subunits, driven by Rous sarcoma virus long terminal repeat promoter.** Cell lysates of the samples, shown in Figure 1D, were separated by SDS-PAGE and immunoblotted with different antibodies. *, non-specific cellular protein, recognized by nsP4 antibody.
(TIF)

Figure S2 **Expression of SFV replicase subunits, driven by hEF1α/HTLV composite promoter.** Cell lysates of the samples, shown in Figure 1E, were separated by SDS-PAGE and immunoblotted with different antibodies. *, non-specific cellular protein, recognized by nsP4 antibody.
(TIF)

Figure S3 **Induction of IFN-β by SFV replicase in MEF cells.** MEFs were transfected by electroporation with dsRNA [poly(I:C)] and plasmid DNA (pRep-RDR and pRep-RDR/GAA) in the presence or absence of chloroquine. At 12 hr post transfection, the amount of IFN-β was determined in the cell culture medium by ELISA. ND, not detectable; CQ, chloroquine. Error bars represent the standard deviation of three experiments.
(TIF)

Figure S4 **Detection of DI-RNA in polyA+ and polyA− RNA fractions purified from SFV4-Rluc and SFV4-Rluc-RDR infected MEF cells.** The RNAs shown in Figure 7A were used as templates for strand-specific reverse transcription followed by PCR. Positive and negative strands of DI-RNAs were reverse-transcribed using the 3′SFV and 5′SFV primers (specified in the Materials and Methods section), respectively. DI-RNA, viral defective interfering RNA; ns, non-specific signal.
(TIF)
