# Supplementary figures and images for: RIG-I and MDA-5 Detection of Viral RNA-dependent RNA Polymerase Activity Restricts Positive-Strand RNA Virus Replication
Source: PLoS Pathog. 2013 Sep 5;9(9):e1003610. doi: 10.1371/journal.ppat.1003610 (PMC3764220; doi:10.1371/journal.ppat.1003610)

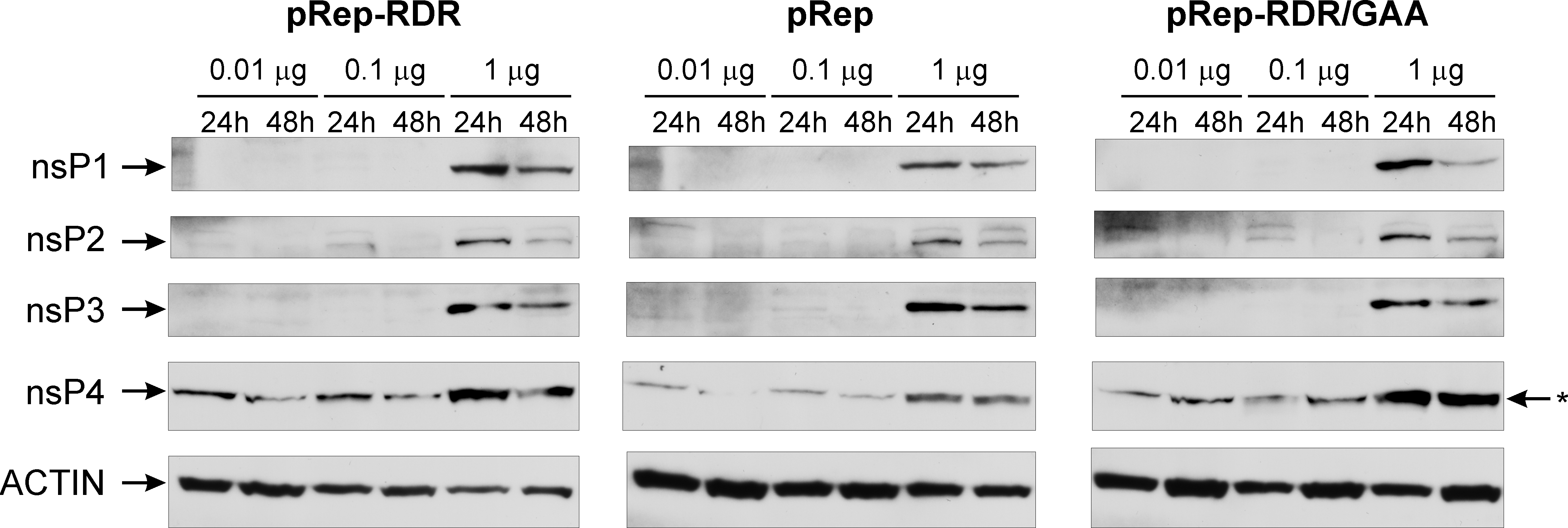

Supplement: Figure S1 — Expression of SFV replicase subunits, driven by Rous sarcoma virus long terminal repeat promoter. Cell lysates of the samples, shown in Figure 1D, were separated by SDS-PAGE and immunoblotted with different antibodies. *, non-specific cellular protein, recognized by nsP4 antibody. (TIF) [file ppat.1003610.s001.tif]

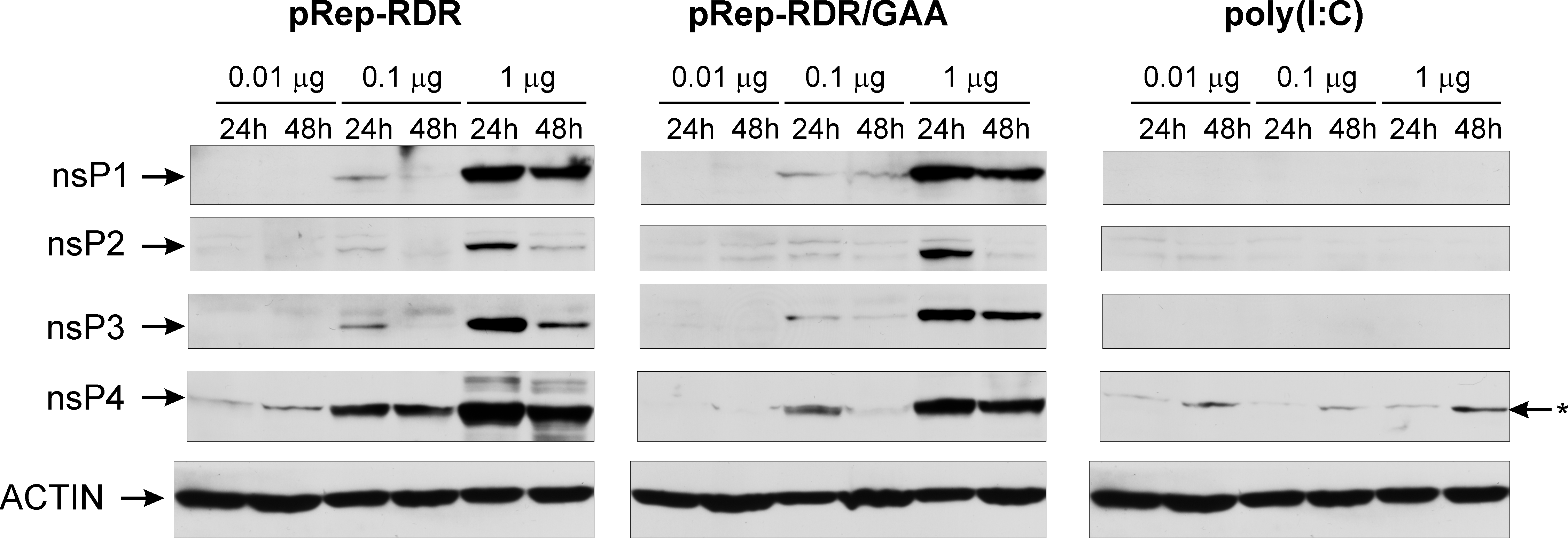

Supplement: Figure S2 — Expression of SFV replicase subunits, driven by hEF1α/HTLV composite promoter. Cell lysates of the samples, shown in Figure 1E, were separated by SDS-PAGE and immunoblotted with different antibodies. *, non-specific cellular protein, recognized by nsP4 antibody. (TIF) [file ppat.1003610.s002.tif]

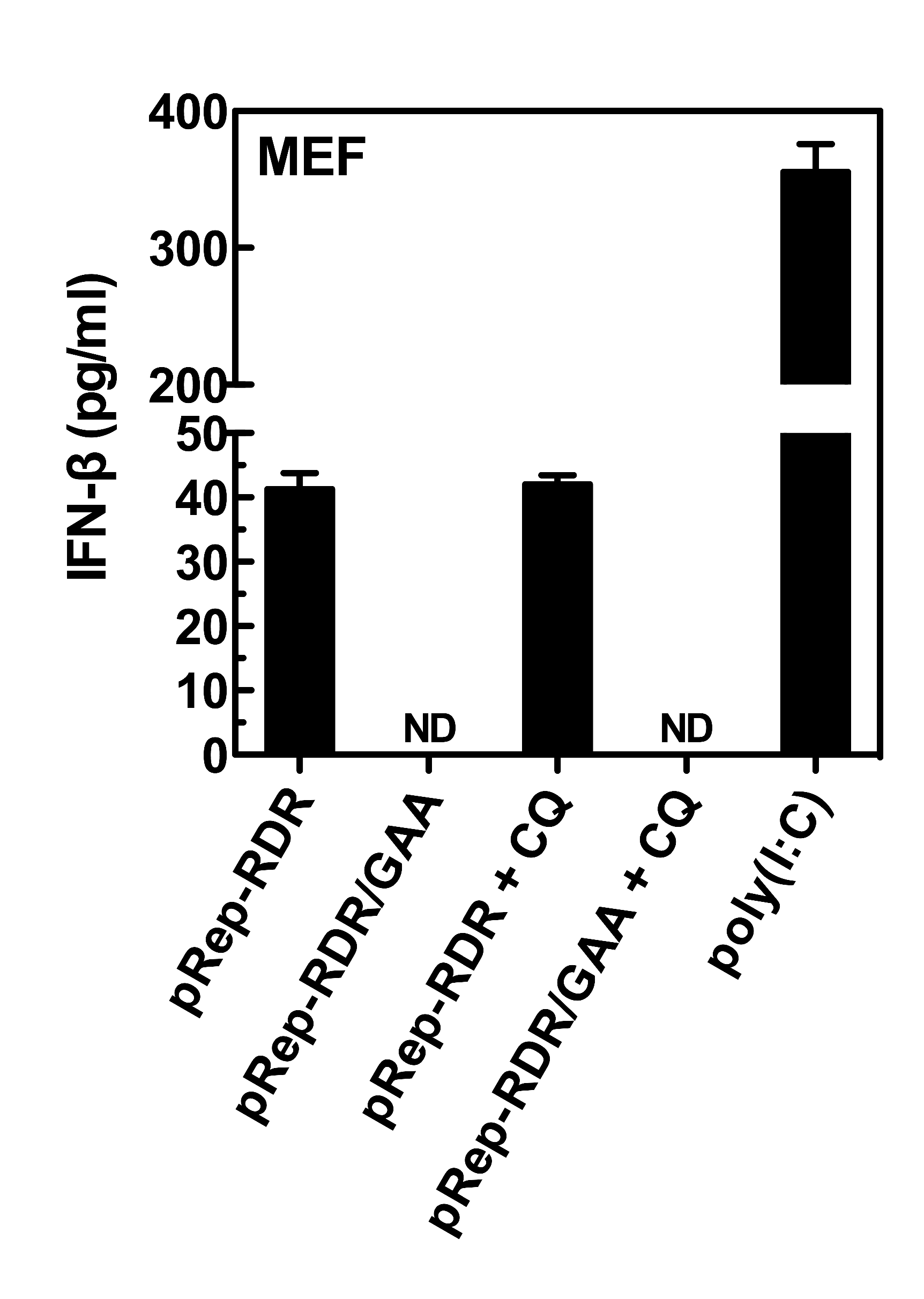

Supplement: Figure S3 — Induction of IFN-β by SFV replicase in MEF cells. MEFs were transfected by electroporation with dsRNA [poly(I:C)] and plasmid DNA (pRep-RDR and pRep-RDR/GAA) in the presence or absence of chloroquine. At 12 hr post transfection, the amount of IFN-β was determined in the cell culture medium by ELISA. ND, not detectable; CQ, chloroquine. Error bars represent the standard deviation of three experiments. (TIF) [file ppat.1003610.s003.tif]

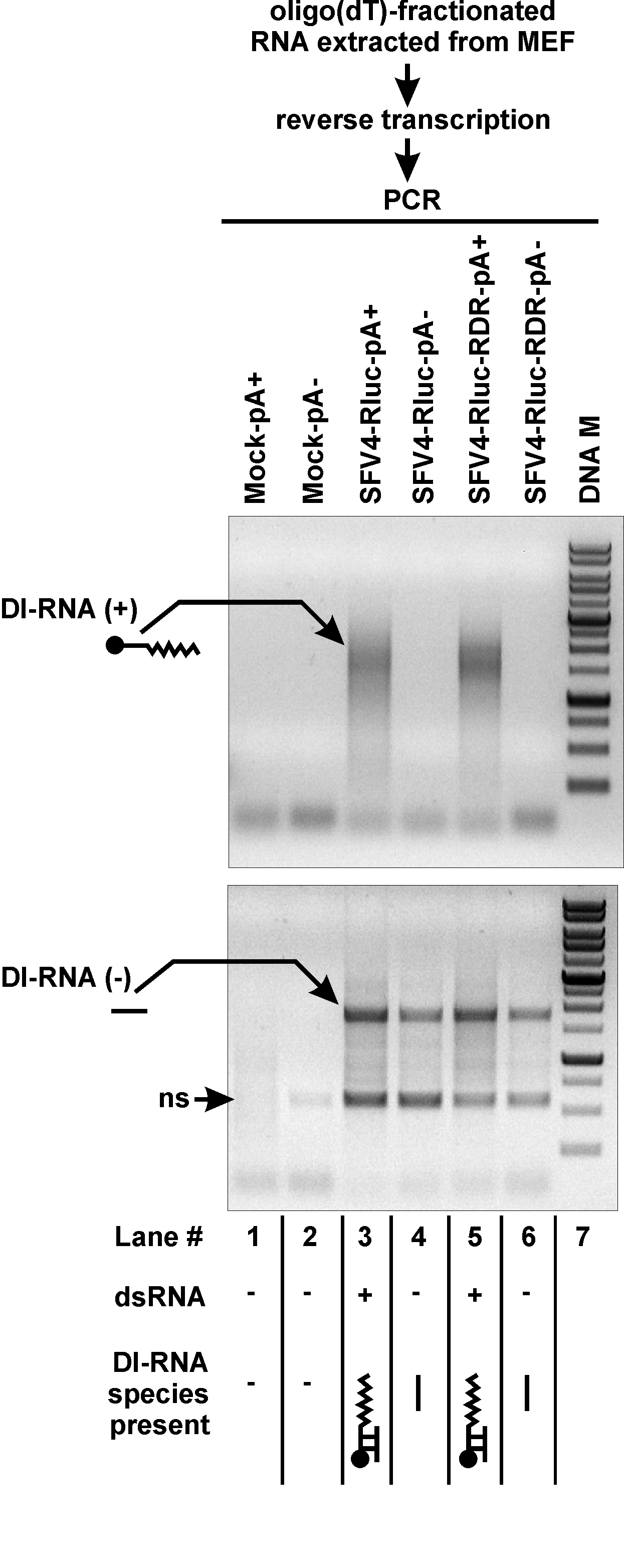

Supplement: Figure S4 — Detection of DI-RNA in polyA+ and polyA− RNA fractions purified from SFV4-Rluc and SFV4-Rluc-RDR infected MEF cells. The RNAs shown in Figure 7A were used as templates for strand-specific reverse transcription followed by PCR. Positive and negative strands of DI-RNAs were reverse-transcribed using the 3′SFV and 5′SFV primers (specified in the Materials and Methods section), respectively. DI-RNA, viral defective interfering RNA; ns, non-specific signal. (TIF) [file ppat.1003610.s004.tif]
